# Targeting KDM4C enhances CD8^+^ T cell mediated antitumor immunity by activating chemokine CXCL10 transcription in lung cancer

**DOI:** 10.1136/jitc-2021-003716

**Published:** 2022-02-04

**Authors:** Xiaohua Jie, Yunshang Chen, Ye Zhao, Xijie Yang, Yingzhuo Xu, Jian Wang, Rui Meng, Sheng Zhang, Xiaorong Dong, Tao Zhang, Kunyu Yang, Shuangbing Xu, Gang Wu

**Affiliations:** Cancer Center, Union Hospital, Tongji Medical College, Huazhong University of Science and Technology, Wuhan, China

**Keywords:** radioimmunotherapy, lung neoplasms, tumor microenvironment

## Abstract

**Background:**

Although immune checkpoint blockade (ICB) has been proven to achieve a persistent therapeutic response in various tumor types, only 20%–40% of patients benefit from this treatment. Radiotherapy (RT) can enhance tumor immunogenicity and improve the ICB response, but the outcome achieved by combining these two modalities remains clinically unsatisfactory. We previously uncovered that lysine-specific demethylase 4C (KDM4C) is a regulator of radiosensitivity in lung cancer. However, the role of KDM4C in antitumor immunity has not yet been investigated.

**Methods:**

Infiltrating immune cells in our mouse tumor model were screened by flow cytometry. An in vivo subcutaneous transplanted tumor model and in vitro conditioned culture model were constructed to detect the quantitative and functional changes in CD8^+^ T cells. RNA sequencing and chromatin immunoprecipitation-PCR assays were used to explore the downstream regulatory mechanism of KDM4C in antitumor immunity. A C57BL/6 mouse tumor model was developed to evaluate the efficacy and safety of a triple therapy (the KDM4C-specific inhibitor SD70 plus RT and an anti-PD-L1 antibody) in lung cancer in vivo.

**Results:**

Genetical or pharmacological inhibition of KDM4C specifically increased CD8^+^ T cell infiltration; promoted the proliferation, migration and activation of CD8^+^ T cells; and alleviated CD8^+^ T cell exhaustion in mouse tumor tissues. Mechanistically, KDM4C inhibition increased the binding of H3K36me3 to the CXCL10 promoter region, thus inducing CXCL10 transcription and enhancing the CD8^+^ T cell mediated antitumor immune response. More importantly, among the tested regimens, the triple therapy achieved the best therapeutic efficacy with tolerable toxicity in lung cancer.

**Conclusions:**

Our data reveal a crucial role for KDM4C in antitumor immunity in lung cancer and indicate that targeting KDM4C in combination with radioimmunotherapy might be a promising synergistic strategy in lung cancer.

## Introduction

The use of immune checkpoint inhibitors has become a powerful treatment strategy in many types of cancer. Immunotherapy is based on activating the immune system against tumor cells and is characterized by its fine specificity and ability to induce immune memory, which can achieve rapid and lasting tumor clearance.^1^ Despite the achievement of remarkable clinical success in several human tumors, the overall sensitivity of patients to immune checkpoint blockade (ICB) is relatively low.^1 2^ The average response rate of ICBs as a monotherapy in patients with different tumor types is less than 30%.^3 4^ Therefore, improving the antitumor efficacy of ICB has become one of the main challenges in clinical application.

There is a growing body of evidence showing that the efficacy of ICB is dependent on a strong antitumor immune response, which is usually impaired in most tumors.^3 5^ According to the immune cell type, tumors can be roughly divided into ‘hot’ (T cell inflammation) or ‘cold’ (T cells absent) tumors. Immunologically ‘hot’ tumors are characterized by the presence of a large number of infiltrating CD8^+^ cytotoxic T cells (CTLs) and antigen-presenting cells (such as dendritic cells (DCs)), and rapid and sustained tumor clearance can often be achieved when tumors with this type of profile are treated with ICBs. In contrast, immunologically ‘cold’ tumors are characterized by a lack of preexisting tumor-infiltrating lymphocytes (TILs), and these tumors can be further subdivided into ‘immune-excluded’ tumors, in which T cells are attracted to the tumor but fail to infiltrate, and ‘immune-desert’ tumors, in which T cell tumor infiltration is absent. Although ICBs aim to boost the body’s immune responses against tumors, immunotherapy in patients without preexisting TILs is rarely effective.^6–8^ Numerous studies have shown that CD8^+^ T cells are the primary mediators of antitumor immunity. Thus, enhancing their infiltration into tumors has always been at the center of immunotherapeutic strategies.^9 10^ Additionally, radiotherapy (RT) has been proven to enhance antitumor immunity by releasing tumor-associated antigens and promoting TIL infiltration into tumors.^11 12^ However, the synergistic effects achieved with RT combined with immunotherapy are still clinically unsatisfactory.

Epigenetics is defined as the dynamic modification of the genome without changing the DNA sequence. Abnormal epigenetic changes usually lead to aberrant gene expression and tumor promotion.^13 14^ At present, epigenetic therapy has been shown to modulate various components of the tumor microenvironment (TME), including increasing tumor-associated antigen expression, enhancing antigen processing and presentation, inducing CD8^+^ T cell infiltration and activation, preventing or reversing T cell exhaustion, and enhancing the abundance of infiltrating effector T cells and/or memory T cells.^15 16^ Therefore, the combination of epigenetic therapy and immunotherapy is considered a promising treatment strategy for cancer.^15 16^ Histone lysine-specific demethylase 4C (KDM4C) can specifically catalyze the demethylation of the substrate molecules H3K9me3 and H3K36me3, which subsequently control the expression of downstream target genes and participate in tumorigenesis.^17–19^ Our previous study found that KDM4C-induced TGF-β2 transcriptional activation mediated by downregulating the enrichment of H3K9me3 at its promoter region enhances Smad/ATM/Chk2 signaling, which leads to radioresistance in lung cancer.^20^ However, the effect and regulatory mechanism of KDM4C in antitumor immunity remain largely unknown.

In this study, we found that KDM4C inhibition enhanced the transcriptional activity of CXCL10 by increasing the concentration of H3K36me3 at its promoter region in lung cancer. This, in turn, stimulated the proliferation, migration, and activation of CD8^+^ T cells and delayed exhaustion in these cells, ultimately promoting antitumor immunity. More importantly, among the tested regimens, a triple therapy (the KDM4C-specific inhibitor SD70 plus RT and an anti-PD-L1 antibody) produced the best antitumor effect against lung cancer in vivo. Our findings provide solid preclinical evidence for the clinical translation of this new treatment strategy.

## Materials and methods

### Cell lines

Human and mouse lung cancer cell lines (A549, H1299, H460 and Lewis) were purchased from American Type Culture Collection and routinely tested to confirm that they were free of mycoplasma and other pathogens. RPMI-1640 or DMEM supplemented with 10% fetal bovine serum and 100 IU/mL penicillin-streptomycin solution was used for limited-generation culture in a humidified incubator containing 5% CO_2_ at 37℃. Lewis cell lines with stable KDM4C knockdown were constructed by using a method described previously.^20 21^ SiRNA transfection was carried out using transfection reagent (Lipofectamine RNAiMAX, Invitrogen) for 48 hours. The shRNA and siRNA sequences used were as follows:

Control shRNA: 5′-TTCTCCGAACGTGTCACGT-3′.

KDM4C shRNA-1: 5′-GCACAAGATGACCCTCATT-3′.

KDM4C shRNA-2: 5′-CCAAGAGCTTACAGGGCAA-3′.

Scramble siRNA: 5′-UUCUCCGAACGUGUCACGU-3′.

SiKDM4C #1: 5′-AGAUAGCAGCAAUGAAGAA-3′.

SiKDM4C #2: 5′-GGCGCCAAGUGAUGAAGAA-3′.^20^

### Antibodies and reagents

For flow cytometry, Zombie NIR (423105), PE/Cy7-conjugated antimouse CD45 (103113), FITC-conjugated antimouse CD3 (100203), PE-conjugated antimouse CD4 (100407), Brilliant Violet 510-conjugated antimouse CD8a (100751), Brilliant Violet 421-conjugated antimouse CD25 (102033), FITC-conjugated antimouse CD11b (101205), FITC-conjugated antimouse CD11c (117305), PE/Cy7-conjugated antimouse F4/80 (123113), PE-conjugated antimouse CD80 (104707), APC-conjugated antimouse CD86 (105011), PE-conjugated antimouse CD206 (141706), PerCP/Cyanine5.5-conjugated antimouse CD19 (115534), APC-conjugated antimouse FOXP3 (17-5773-82, eBioscience), APC-conjugated antimouse Ki-67 (652405), and Brilliant Violet 421-conjugated antimouse IFN-γ (505829), Brilliant Violet 421-conjugated antimouse granzyme B (GZMB) (recombinant antibody, 396414), APC-conjugated antimouse Perforin (154304), PE-conjugated antimouse CD107a (328608), and APC-conjugated antimouse CD39 (143810) were used. All the previously mentioned antibodies were purchased from BioLegend unless otherwise stated and used according to the antibody instructions. For western blotting, rabbit anti-KDM4C (1:800, A300-885A, Bethyl Laboratories), mouse anti-CXCL10 (1 µg/mL, AF-466-NA, R&D Systems), rabbit anti-H3K9me3 (1:800, Ab8898, Abcam), rabbit anti-H3K36me3 (1:800, Ab9050, Abcam), rabbit anti-H3 (1:1000, Ab1791, Abcam), rabbit anti-STING (1:1000, 19 851–1-AP, Proteintech), rabbit anti-MAVS (1:1000, 66 911–1-Ig, Proteintech), rabbit anti-IRF3 (1:1000, 11 312–1-AP, Proteintech), rabbit anti-TLR3 (1:1000, A11778, ABclonal), and mouse anti-GAPDH (1:3000, AC033, ABclonal) antibodies were used. For chromatin immunoprecipitation (ChIP) assays, rabbit anti-H3K36me3 (5 µg, Ab9050, Abcam) was used. For immunohistochemistry, anti-CD8 (10 µg/mL, NBP1-49045SS, NOVUS) was used. The following additional reagents were purchased: SD70 (Xcess Biosciences, M60194Mub), CSFE (21888, Sigma-Aldrich), CD8 (TIL) MicroBeads (130-116-478, Miltenyi), CD3/CD28 Dynabeads (11 452D, Invitrogen), IL2 (HY-P7077, MCE), an anti-PD-L1 monoclonal antibody (10F.9G2, BioXCell), an anti-CD8 neutralizing antibody (clone 2.43; BioXCell) and an anti-CXCL10 neutralizing antibody (MAB266, R&D Systems).

### Tumor models and in vivo treatments

Our subcutaneously transplanted tumor mouse model has been described in detail previously.^20 22^ Female C57BL/6 mice were randomly divided into the indicated groups and subcutaneously injected with 1×10^6^ Lewis cells (control cells or stable KDM4C-knockdown Lewis cells). Tumor volume was measured every 3 days, and the formula used was volume (V)=length (L)×width (W)^2^/2. For SD70 therapy, SD70 drug preparation and the experimental dose have been described in previous studies.^20 23^ A dose division mode of 8 Gy ×3 was used for RT. For PD-L1 blockade, an anti-PD-L1 monoclonal antibody was injected intraperitoneally every 4 days at a dose of 10 mg/kg. For depletion experiments, 200 µg anti-CD8 neutralizing antibody was injected i.p. every 4 days. Anti-CXCL10 neutralizing antibody was used according to a corresponding schedule and dose as indicated.

### Flow cytometry

Tumor tissues were cut into pieces and made into single cell suspension. The reagents used for staining were as follows: Zombie NIR was used to label dead cells; anti-CD45, anti-CD3, anti-CD4, and anti-CD8 antibodies were used to label T cells; anti-CD45, anti-CD4, anti-CD25 and anti-FOXP3 antibodies were used to label regulatory T (Treg) cells; anti-CD11b, anti-F4/80, anti-CD86, and anti-CD206 antibodies were used to label macrophages; anti-CD11c, anti-CD86, and anti-CD80 antibodies were used to label DCs; anti-CD45 and anti-CD19 antibodies were used to label B cells; and markers of CD8^+^ T cells (Ki67, IFN-γ, GZMB, Perforin, CD107a, PD-1, and CD39) were also stained. Staining for surface markers was performed in the dark at 4℃ for 30 min. Intracellular markers (FOXP3, CD206, Ki67, IFN-γ, GZMB, and Perforin) were identified by fixation, permeabilization, and staining according to the antibody instructions. All samples were detected on a CytoFLEX flow cytometer (Beckman Kurt Trading Co, Ltd, USA), and flow cytometry data were analyzed.

### Construction of an in vitro conditioned culture model

The supernatant of Lewis cells with stable KDM4C knockdown or treated with SD70 (1.5 µM) for 24 hours was filtered using 0.22 µm aseptic filters. Preparation of single cell suspension from spleen of C57BL/6 mice. Each 100 µL cell suspension was incubated with 10 µL Miltenyi CD8 (TIL) MicroBeads in the dark at 4℃ for 15 min, MACS Buffer (130-091-222, Miltenyi Biotec) was added twice for washing, and the cells were resuspended in 1 mL buffer. Naive CD8^+^ T cells were separated with a MiniMACS Separator and cultured with a prepared cell supernatant. CD3/CD28 Dynabeads (2 µL/well) and IL2 (20 ng/mL) were added for stimulation, and a functional experiment was carried out 3 days later.

### CD8^+^ T cell proliferation assays

The proliferative ability of CD8^+^ T cells was evaluated by detecting the expression of Ki67 on the surface of CD8^+^ T cells and by CFSE staining. An antimouse Ki-67 antibody was added to a CD8^+^ T cell suspension and incubated in the dark for 30 min. After washing, the expression of Ki67 was detected by flow cytometry. For the CFSE staining experiment, naive CD8^+^ T cells were incubated with CFSE dye at 4℃ for 8 min. After washing, the CD8^+^ T cells were resuspended in conditioned medium and seeded in a 96-well plate (100 µL/well contained 2500 CD8^+^ T cells), and CD3/CD28 Dynabeads (2 µl/well) and IL2 (20 ng/mL) were added to each well. After 3 days, the cells were collected, and the proportion of CFSE-positive cells was detected by flow cytometry.

### CD8^+^ T cell mediated cytotoxicity assay

Activated CD8^+^ T cells obtained from the in vitro conditioned culture model and Lewis cells were seeded in 96-well plates or 24-well plates at the indicated ratio and cocultured with conditioned medium for 24 hours. Then, the T cells were thoroughly washed and removed using phosphate-buffered saline, and the surviving tumor cells were fixed and stained with crystal violet. Surviving tumor cells were also stained with CCK-8, and the killing rate was calculated according to the following formula: specific lysis = (% lysis − % nonspecific lysis)/(% maximum lysis − % nonspecific lysis). Cocultured tumor cells were stained for apoptosis markers, and the proportion of apoptotic cells was detected by flow cytometry.

### In vivo and in vitro T cell migration assays

An in vitro migration experiment was carried out in a Transwell system, which included a polycarbonate membrane with a 5 mm pore size. Activated CD8^+^ T cells were washed twice, resuspended in serum-free medium and then added to the top of the chamber, and conditioned medium was added to the bottom of the chamber. After 24 hours of culture, the cells at the bottom of the chamber were collected and fixed with 4% paraformaldehyde. The number of CD8^+^ T cells passing through the membrane was quantified by running on CytoFLEX flow cytometer for 30 s. For the in vivo T cell migration experiment, pretreated activated CD8^+^ T cells (1×10^6^) were injected into the tail vein of tumor-bearing mice. Forty-eight hours later, the infiltration of CD8^+^ T cells in the tumor tissue was detected by immunohistochemical staining.

### Biochemical evaluation of the peripheral blood

After anesthesia administration, the peripheral blood of mice was obtained by retro-orbital puncture, collected and thoroughly shaken immediately in a heparin anticoagulant tube. The supernatant was absorbed after centrifugation (5000 rpm/5 min). An MNCHIP automatic biochemical analyzer (Pointcare M4) was used to detect common biochemical indexes in the peripheral blood of mice.

### RNA-seq

Lewis cells were treated with DMSO or SD70 for 24 hours. All the samples were sequenced by BGI-Shenzhen (China). The cDNA library was constructed using the BGISEQ-500 platform. After the clean reads were obtained, they were compared with a reference genome sequence (reference genome: GCF_000001635.26_GRCm38.p6) by HISAT, and the differentially expressed genes were obtained for follow-up analysis. The RNA-seq data in this study were deposited in the Gene Expression Omnibus database under the accession number GSE178177 (https://www.ncbi.nlm.nih.gov/geo).

### Real-time quantitative PCR (qRT-PCR)

qRT-PCR was performed on a StepOnePlus real-time PCR system (Thermo Fisher Scientific) using Genious 2×SYBR Green Fast qPCR Mix (RK21204, ABclonal). The experiment was repeated three times independently, with GAPDH as the control gene. The sequences of the primers used are listed in online supplemental table S1.

10.1136/jitc-2021-003716.supp1 Supplementary data

### Western blotting

This method has been described in detail in our previous studies.^20 21^ Briefly, cells were added to NETN buffer to prepare cell lysates, incubated at 4℃ for 20 min and centrifuged at 12 000 rpm for 10 min to obtain cellular proteins. Then, SDS-PAGE and western blotting were performed.

### ELISA

The level of CXCL10 secreted into the supernatant was measured using the Mouse CXCL10 ELISA Kit (DY466-05, R&D Systems) and Human CXCL10 ELISA Kit (JL28903, Jianglai Biological) according to the manufacturer’s instructions.

### ChIP-PCR

The ChIP assay component was performed as previously described.^20^ Briefly, we used an EZ-Chip Chromatin immunoprecipitation kit (Millipore) according to the manufacturer’s instructions. RT-PCR was used to detect changes in H3K36me3 accumulation at the promoter region of CXCL10. The sequences of the primers used are listed in online supplemental table S2.

### Immunohistochemistry and H&E staining

We performed immunohistochemistry assays as described previously.^20^ Briefly, we randomly selected three different visual fields in each sample and used ImageJ software to count CD8^+^ T cells. For H&E staining, mouse tissues were fixed with 4% paraformaldehyde, embedded in paraffin, fully dewaxed, hydrated, and stained with H&E. A visual field was randomly selected under a light microscope to observe the pathomorphological changes in each mouse tissue sample.

### Statistical analysis

All experiments were repeated at least three times independently. All quantitative data are presented as the mean±SD unless otherwise stated. A survival curve was drawn according to the Kaplan-Meier method. Differences between groups were compared by an unpaired two-tailed t-test. P<0.05 was consider to indicate a significant difference (*p<0.05, **p<0.01, ***p<0.001).

## Results

### Genetical or pharmacological inhibition of KDM4C enhances the infiltration and migration of CD8^+^ T cells

**Figure 1:**
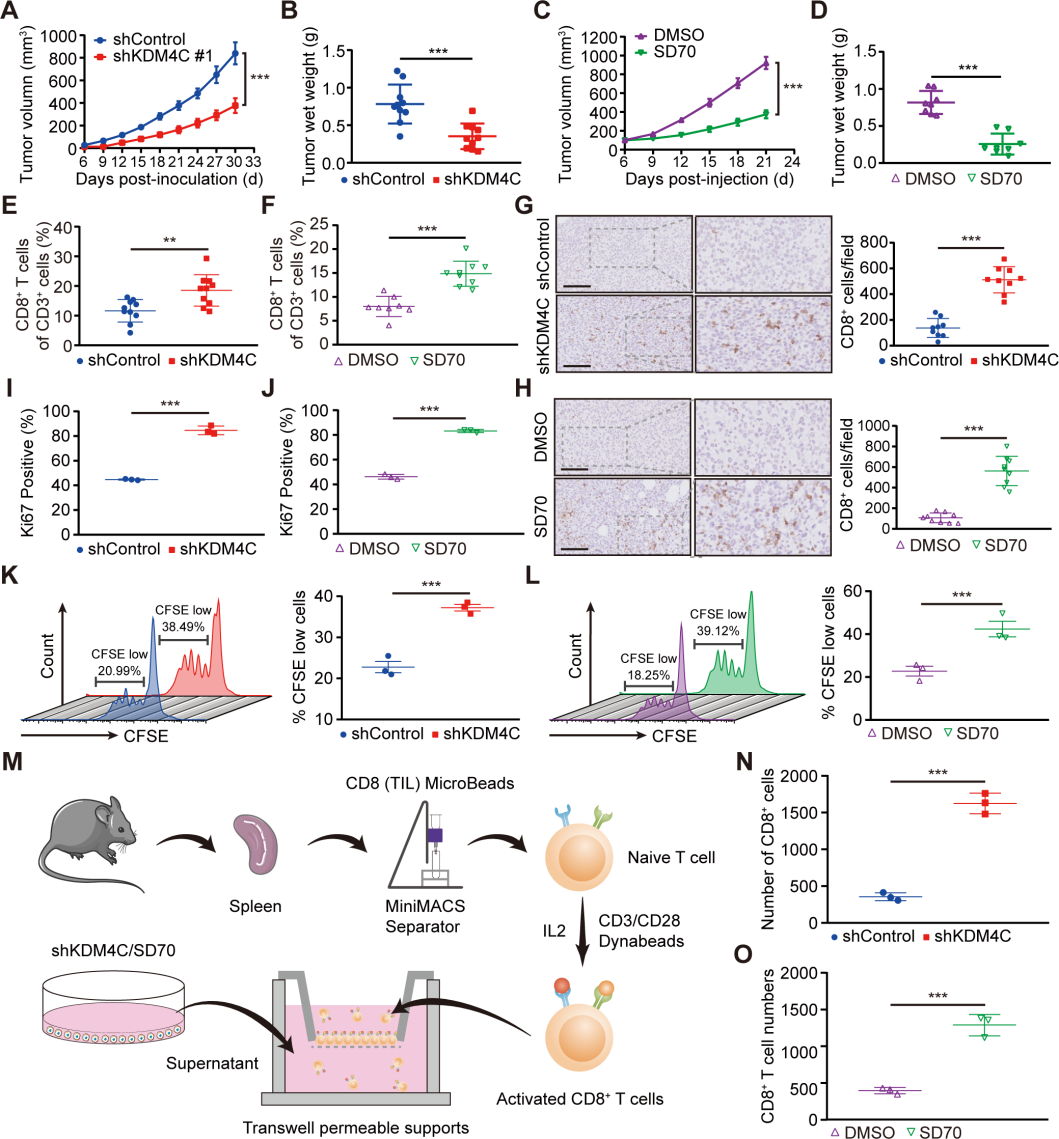
KDM4C inhibition enhances CD8^+^ T cell infiltration and migration. (A) C57BL/6 mice were injected subcutaneously with 1×10^6^ stable Lewis cells (sh-control or sh-KDM4C). The volumes of the subcutaneously transplanted tumors were measured every 3 days, and the growth curves were drawn (mean±SEM; n=10; ***p<0.001). (B) All subcutaneously transplanted tumor weights were measured (n=10/group, ***p<0.001). (C) Mice bearing Lewis tumors were treated with SD70 (10 mg/kg) when the tumor volume reached a calculated average of 100 mm^3^. After administration for five consecutive days, SD70 was administered once every 3 days (mean±SEM; n=8; ***p<0.001). (D) All subcutaneously transplanted tumor weights of each group (n=8/group, ***p<0.001). (E, F) The percentages of CD8^+^ T cells in CD3^+^ cells in tumor tissues after the indicated treatment. The mean±SD is shown. **P<0.01, ***p<0.001. (G, H) Left panel: representative immunohistochemical staining images of CD8^+^ T cells in CD3^+^ cells in tumor tissues after the indicated treatment. Scale bar: 100 µm. Right panel: CD8^+^ T cells counted with ImageJ software. Three fields were selected for each sample. ***p<0.001. (I, J) The percentages of proliferating CD8^+^ T cells were analyzed by Ki67 staining. The mean±SD is shown. (K, L) At 72 hours after CFSE staining, the proliferation of CD8^+^ T cells was measured by flow cytometry. ***P<0.001. (M) Schematic diagram of in vitro CD8^+^ T cell migration assays. (N, O) The number of CD8^+^ T cells passing through the membrane of a Transwell system was calculated by flow cytometry. The mean±SD is shown. ***P<0.001.

To investigate the effects of KDM4C inhibition on antitumor immunity, we used shRNA technology to stably knockdown the expression of KDM4C or used the specific KDM4C inhibitor SD70 in in vivo experiments. We found that the genetical or pharmacological inhibition of KDM4C significantly reduced the growth of Lewis cells in vitro and subcutaneously transplanted lung cancer tumors in C57BL/6 mice (figure 1A–D and online supplemental figure S1). Flow cytometry analysis of the tumor immune landscape in transplanted tumors revealed that KDM4C inhibition could significantly increase the proportion of CD8^+^ T cells (figure 1E, F and online supplemental figure S2). Interestingly, other immune cells, such as Treg cells, B cells, DCs, and macrophages, showed no significant differences (online supplemental figure S3). Similarly, immunohistochemical analysis showed that the infiltration of CD8^+^ T cells in the KDM4C inhibition group was significantly increased compared with that in the control group (figure 1G, H).

10.1136/jitc-2021-003716.supp2 Supplementary data

10.1136/jitc-2021-003716.supp3 Supplementary data

10.1136/jitc-2021-003716.supp4 Supplementary data

**Figure 2:**
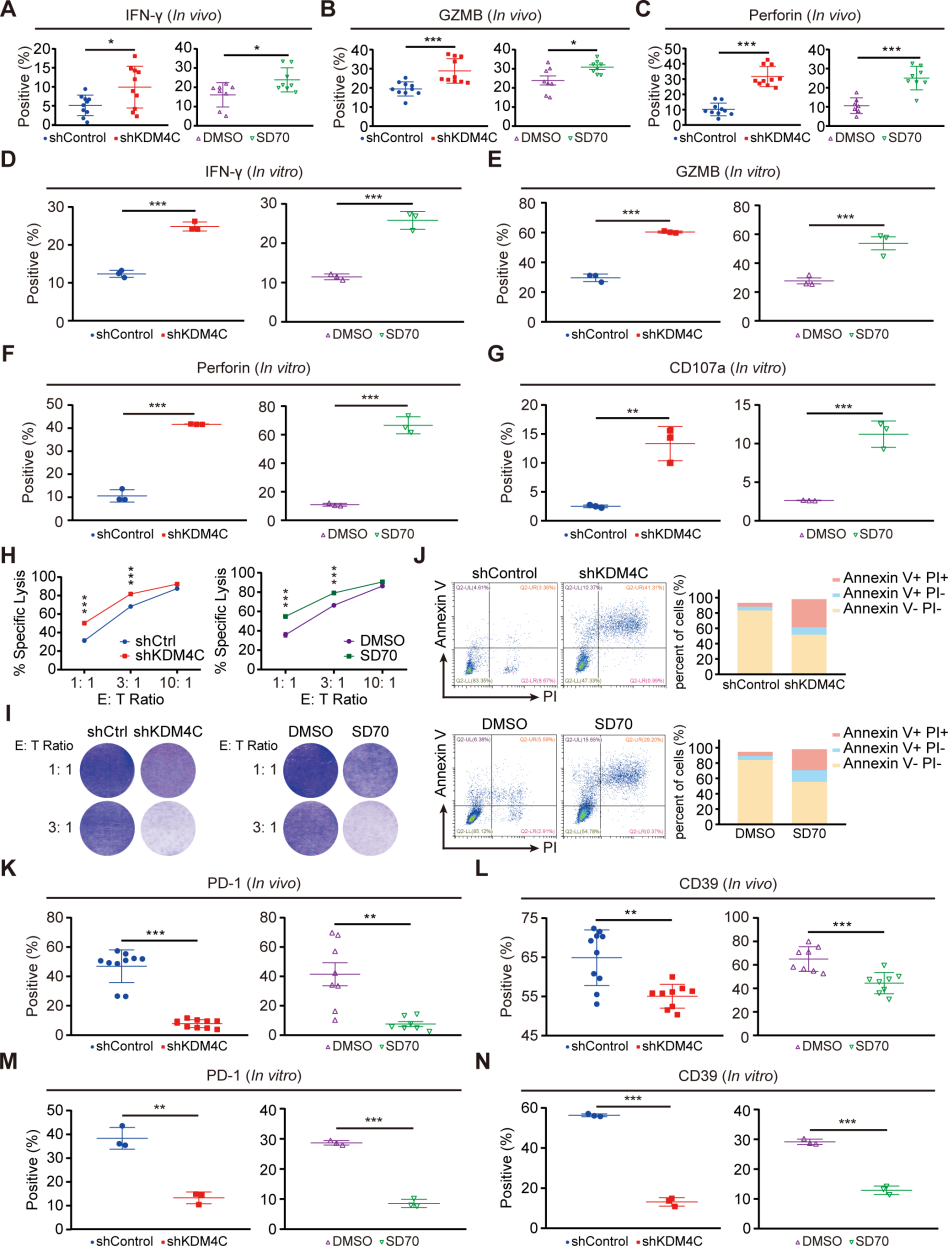
KDM4C inhibition augments antitumor immunity and delays exhaustion in CD8^+^ T cells in vitro and in vivo. (A–C) CD8^+^ T cells isolated from tumor tissues after the indicated treatment were stimulated with PMA (100 ng/mL), monensin sodium salt (1 µg/mL), and ionomycin (100 ng/mL) for 6 hours, and cell cytotoxicity markers (IFN-γ, GZMB and Perforin) were assessed by flow cytometry. *P<0.05, ***p<0.001. (D–G) Evaluation of the effect of KDM4C silencing on the cytotoxicity of CD8^+^ T cells in an in vitro conditioned culture model. ***P<0.001. (H) Specific lysis of Lewis tumor cells by CD8^+^ T cells pretreated with the indicated conditioned medium. Each experiment was repeated three times independently. ***P<0.001. (I) Lewis cells were cocultured with pretreated CD8^+^ T cells at the indicated proportion for 24 hours, and the T cells were washed and removed with phosphate-buffered saline, fixed with 4% paraformaldehyde and stained with crystal violet. Each experiment was performed in triplicate. (J) Lewis cells and pretreated CD8^+^ T cells were cocultured at 1:3. After 24 hours, the tumor cells were stained for an apoptosis assay, and the proportion of apoptotic Lewis cells was detected by flow cytometry. (K, L) After the indicated treatment, the levels of the exhaustion markers PD-1 and CD39 on CD8^+^ T cells in tumor tissues were assessed. **P<0.01, ***p<0.001. (M, N) Flow cytometry analyses for exhaustion markers on CD8^+^ T cells treated with the indicated conditioned medium are shown. **P<0.01, ***p<0.001.

To further investigate the effect of KDM4C on the function of CD8^+^ T cells, we constructed an in vitro conditioned culture model. As shown in online supplemental figure S4A, individual mouse spleens were processed into single-cell suspensions and incubated with CD8 (TIL) MicroBeads, and naive CD8^+^ T cells were purified using a MiniMACS Separator system from Miltenyi Biotec. The percentage of purified CD8^+^ T cells detected by flow cytometry was 99.12% (online supplemental figure S4B). Purified CD8^+^ T cells were then seeded in 96-well round-bottom plates and cultured in supernatants from pretreated Lewis cells containing IL2 (20 ng/mL) and CD3/CD28 Dynabeads for 3 days. Flow cytometry analysis revealed significantly increased expression of Ki67 on CD8^+^ T cells after KDM4C inhibition (figure 1I, J). Furthermore, CFSE staining analysis showed that the proportion of CFSE low T cells in the treatment group was significantly higher than that in the control group (figure 1K, L), indicating that KDM4C inhibition could increase the proliferation of CD8^+^ T cells. As shown in figure 1M–O, an in vitro migration experiment showed that the migratory ability of CD8^+^ T cells was significantly improved following KDM4C inhibition. These findings demonstrate that KDM4C suppression increases CD8^+^ T cell tumor infiltration and migration.

10.1136/jitc-2021-003716.supp5 Supplementary data

### KDM4C inhibition augments antitumor immunity and hampers CD8^+^ T cell exhaustion development in vitro and in vivo

CD8^+^ T cells are the main cytotoxic immune cells in tumors and have the abilities to promote antitumor immunity and induce tumor cell death by secreting cytotoxic molecules, such as IFN-γ, GZMB, Perforin, and CD107a.^24^ To clarify the effect of KDM4C on CD8^+^ T cells, we analyzed the expression levels of cytotoxicity markers (IFN-γ, GZMB, Perforin, and CD107a) on tumor-infiltrating CD8^+^ T cells. As shown in figure 2A–C, the expression of cytotoxic molecules in tumor CD8^+^ T cells was significantly upregulated after genetical or pharmacologic inhibition of KDM4C, indicating that KDM4C inhibition promotes the activation of CD8^+^ T cells. Similarly, in vitro experiments also showed increased CD8^+^ T cell activation (figure 2D–G).

To further evaluate the cytotoxic effects of CD8^+^ T cells, we analyzed the killing activity of T cells in vitro. As expected, adding conditioned medium in which KDM4C was inhibited enhanced CD8^+^ T cell mediated tumor cell lysis (figure 2H). Fixation and staining of surviving tumor cells showed that inhibition of KDM4C significantly enhanced the proportion of tumor cells killed by T cells (figure 2I). Additionally, the proportion of apoptotic Lewis cells in the KDM4C intervention group was significantly increased compared with that in the control group (figure 2J). It has been documented that exhausted CD8^+^ T cells, which express various inhibitory receptors, including PD-1 and CD39, are unable to effectively kill tumor cells.^25 26^ To that end, we evaluated the exhaustion state of CD8^+^ T cells in vivo and in vitro and found that the expression of exhaustion markers on CD8^+^ T cells was significantly decreased in the treatment group compared with the control group (figure 2K–N). These results suggest that KDM4C inhibition enhances CD8^+^ T cell mediated antitumor immunity and delays CD8^+^ T cell exhaustion.

### Inhibition of KDM4C increases the binding of the transcriptional activation marker H3K36me3 to the CXCL10 promoter in lung cancer

**Figure 3:**
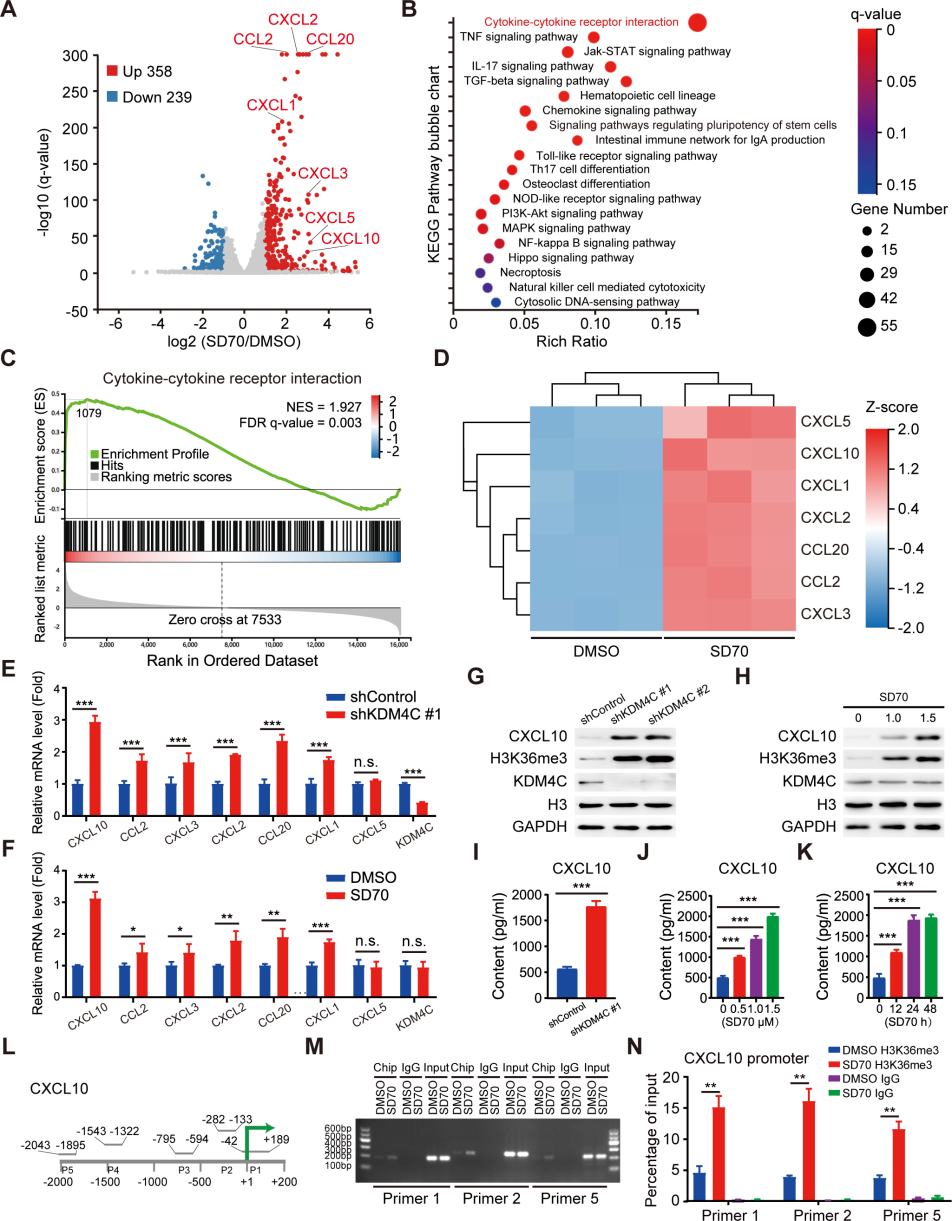
KDM4C inhibition increases CXCL10 expression by promoting transcriptional activation marker H3K36me3 accumulation at the CXCL10 promoter. (A) The volcano plot shows that differentially expressed genes affected by SD70 were related to the cytokine–cytokine receptor interaction. (B) Bubble chart of signaling pathways affected by SD70. According to classification by KEGG pathway annotations, enrichment analysis was carried out using the phyper function, and the p value was calculated and corrected by the FDR to obtain the q-value function. A q value ≤0.05 was usually regarded as indicating significant enrichment. (C) GSEA of the cytokine-cytokine receptor interaction pathway. (D) Heatmap showing chemokine changes after SD70 treatment. (E, F) The mRNA expression of the indicated genes after the indicated treatment was measured by real-time PCR. Statistical analyses by a t-test. *P<0.05, **p<0.01, ***p<0.001 (n=3). (G, H) Western blotting showing that the protein levels of CXCL10 were increased in KDM4C-silenced Lewis cells (n=3). (I–K) ELISA analysis of CXCL10 expression in shKDM4C Lewis cells compared with control cells (I), cells treated with the indicated concentration of SD70 treatment for 24 hours (J), or cells treated for the indicated time with 1.5 µM SD70 (K). ***P<0.001. (L) Sketch map of the ChIP primer design from −2000 to +200 bp around the transcription start site (TSS). (M) Representative gel electrophoresis result images. The experiment was repeated three times. (N) H3K36me3 levels at the CXCL10 promoter were normalized to the input (mean±SEM; n=3; **p<0.01). ChIP, chromatin immunoprecipitation; NES, normalized enrichment score.

To determine the molecular mechanism of KDM4C-induced CD8^+^ T cell activation, we carried out RNA-seq and found that 597 genes were significantly differentially expressed following SD70 treatment (p<0.01, Log2 ≥1) (figure 3A). Further bioinformatic analysis revealed that the cytokine–cytokine receptor interaction pathway was significantly enriched (figure 3A–C), including seven chemokines that exhibited more than twofold enrichment (figure 3D). Six candidate genes with consistent changes (CXCL10, CCL2, CXCL3, CXCL2, CXCL20, and CXCL1) were validated by qRT-PCR, with the chemokine CXCL10 showing the most notable change (figure 3E, F).

Previous studies have shown that CXCL10 is a critical factor in mediating T cell chemotaxis and functional activation^27 28^; thus, we speculated CXCL10 might be a possible key downstream candidate molecule for KDM4C. To test this hypothesis, we first targeted KDM4C genetically or pharmacologically in multiple human lung cancer cell lines and Lewis cells and detected a significant increase in the protein expression of CXCL10 (figure 3G, H and online supplemental figure S5A). Moreover, ELISA analysis showed that KDM4C inhibition promoted exocrine CXCL10 accumulation in a time-dependent and concentration-dependent manner (figure 3I–K and online supplemental figure S5B). It is well established that the decrease in H3K36me3 level mediated by KDM4C often leads to transcriptional inactivation of target genes.^19^ To determine whether KDM4C can directly regulate the expression of CXCL10, we performed ChIP-PCR analysis and found that the enrichment level of H3K36me3 in the promoter region of the CXCL10 gene increased significantly after SD70 treatment (figure 3L–N). In addition, it has been reported that the STING signal pathway and IFN-α are involved in T cell activation and function.^29 30^ Our results showed that STING, MAVS, TLR3 and IRF3 levels remained constant when KDM4C was depleted in A549 and Lewis tumor cells (online supplemental figure S6), suggesting that STING signal pathway and IFN-α are not involved in KDM4C-mediated biological processes in lung cancer. Together, our results suggest that KDM4C controls the expression of CXCL10 by promoting the accumulation of H3K36me3 at the CXCL10 promoter region.

10.1136/jitc-2021-003716.supp6 Supplementary data

10.1136/jitc-2021-003716.supp7 Supplementary data

### KDM4C inhibition promotes antitumor immunity via CXCL10 in lung cancer

**Figure 4:**
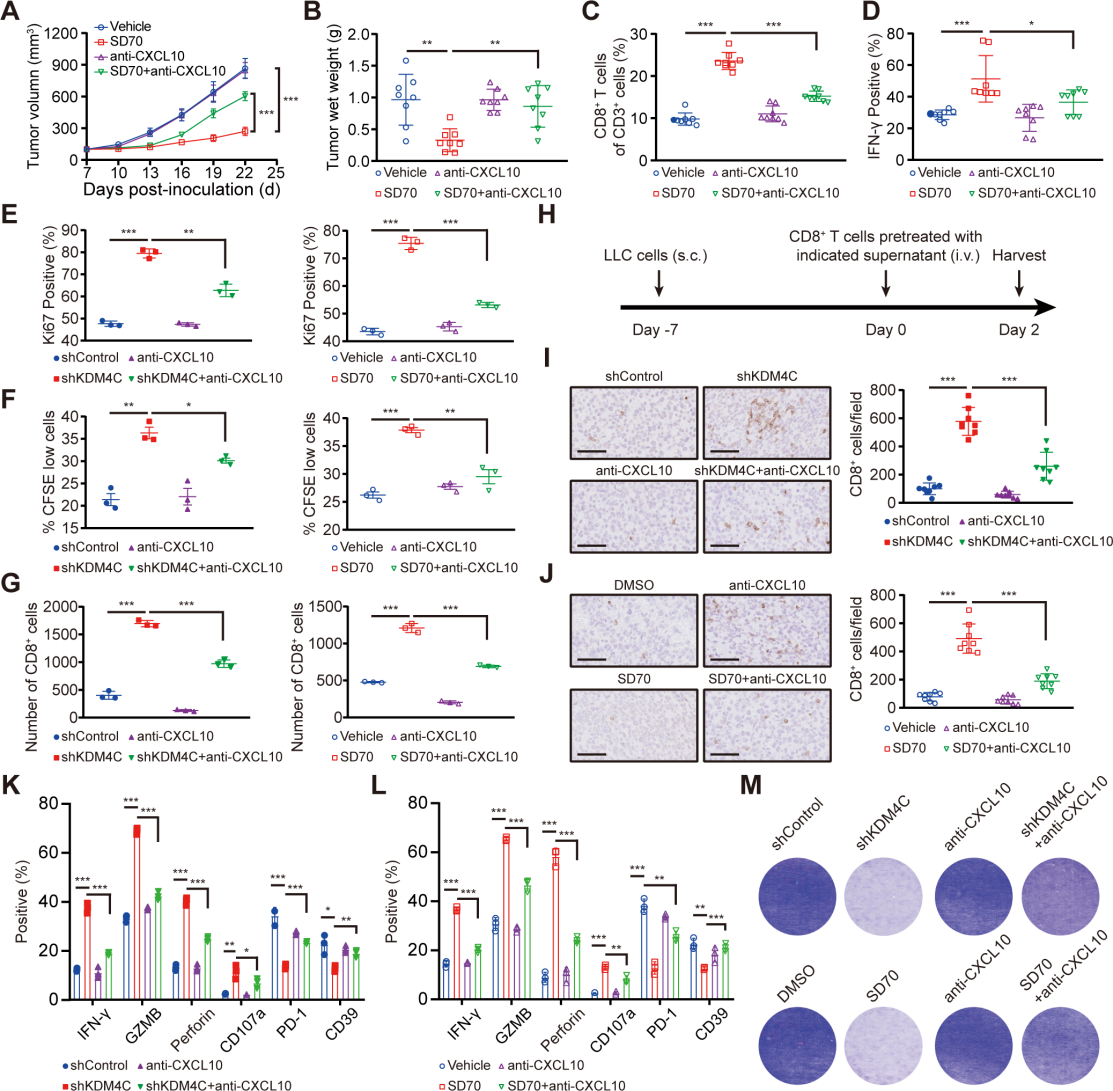
Enhanced antitumor immunity caused by KDM4C inhibition is mediated by CXCL10. (A) C57BL/6 mice were injected subcutaneously with 1×10^6^ Lewis cells. When the tumor volume reached an average of 100 mm^3^, the mice were given a vehicle either alone or in combination with SD70 and/or anti-CXCL10. Tumor volume was measured every 3 days (mean±SEM; n=8; ***p<0.001; two-tailed t-test). (B) Tumor wet weights at 22 days’ postinoculation. n=8, **p<0.01. (C, D) Flow cytometry analyses showing the changes in the percentages of CD8^+^ T cells (C) and IFN-γ^+^ CD8^+^ T cells (D) in each group. n=8/group, *p<0.05, ***p<0.001. (E) Changes in Ki67^+^ CD8^+^ T cells with treatment. n=3, **p<0.01, ***p<0.001. (F) CFSE staining of proliferating CD8^+^ T cells measured by flow cytometry. Each experiment was repeated three times independently. (G) Changes in the in vitro migration of CD8^+^ T cells. n=3, ***p<0.001. (H) Schematic illustration of the in vivo migration experiment. (I, J) IHC was used to analyze the infiltration of CD8^+^ T cells in subcutaneously transplanted tumors for each group. Scale bar: 100 µm; n=8/group; ***p<0.001. (K, L) Flow cytometry was used to detect the expression of cytotoxicity markers (IFN-γ, GZMB, Perforin, and CD107a) and exhaustion markers (PD-1 and CD39) in CD8^+^ T cells in each group. n=3; *p<0.05, **p<0.01, ***p<0.001. (M) Lewis cells in 24-well plates were cocultured with pretreated CD8^+^ T cells in the absence or presence of an anti-CXCL10 neutralizing antibody or control antibody for 24 hours. Crystal violet staining identified the surviving tumor cells. Each group of experiments was carried out three times.

To explore the role of the chemokine CXCL10 in the KDM4C-mediated CD8^+^ T cell immune response, C57BL/6 mice were inoculated subcutaneously with 1×10^6^ Lewis cells and randomly divided into four groups. When the tumor volume reached an average of 100 mm^3^, the mice were given a vehicle either alone or combined with SD70 and/or an anti-CXCL10 neutralizing antibody. All mice were sacrificed on day 22 postinoculation. The growth curves of the transplanted tumors were drawn, and the number and activation state of CD8^+^ T cells in the mouse tumors were analyzed by flow cytometry. As shown in figure 4A–D, blocking CXCL10 partially eliminated the slow growth of subcutaneously transplanted tumors and the increased infiltration and functional activation of CD8^+^ T cells in tumors induced by SD70 treatment.

In addition, conditioned medium obtained from Lewis cells treated with KDM4C-specific shRNA or SD70 for 24 hours was used to culture purified CD8^+^ T cells with or without the anti-CXCL10 neutralizing antibody. We showed that KDM4C inhibition could promote the proliferation of CD8^+^ T cells in vitro, while the anti-CXCL10 neutralizing antibody could partially eliminate this change (figure 4E, F). T cell migration assays performed in vitro and in vivo also found that the anti-CXCL10 neutralizing antibody could partially interfere with the increase in CD8^+^ T cell migration induced by KDM4C inhibition (figure 4G–J). Furthermore, KDM4C inhibition enhanced the expression of activated T cell cytotoxicity markers (IFN-γ, GZMB, Perforin, and CD107a), reduced the expression of exhaustion markers (PD-1 and CD39) and promoted the cytotoxicity of T cells, while these effects could be partially eliminated by the anti-CXCL10 neutralizing antibody (figure 4K–M). These data support the notion that KDM4C inhibition exerts a promotive effect on antitumor immunity in a CXCL10-dependent manner in lung cancer.

### Triple therapy using SD70 and RT and an anti-PD-L1 antibody achieves the best antitumor efficacy with tolerable toxicity in lung cancer

**Figure 5:**
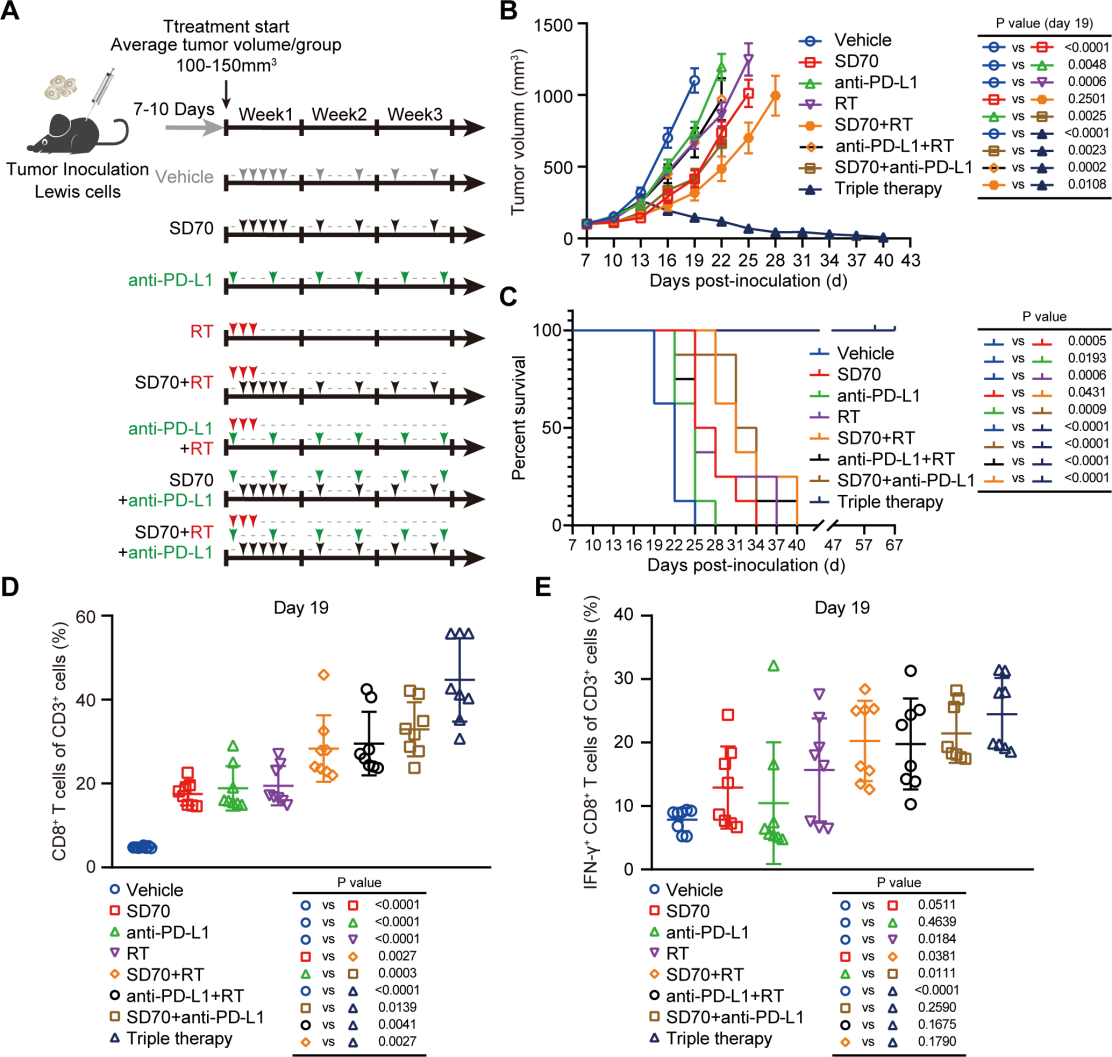
Triple therapy shows the greatest antitumor effects in lung cancer. (A) Schematic illustration of treatment schedule. (B) A C57BL/6 murine model was established, and the mice were randomly divided into eight groups. Each group was treated according to the schedule shown in (A). All subcutaneously transplanted tumors were measured every 3 days with Vernier calipers. When the subcutaneously transplanted tumors in the group reached 1500 mm^3^ or more than 60 days had elapsed from tumor cell injection, the experiment was terminated. The tumor growth curves of subcutaneously transplanted tumors were drawn, and the data on the 19th day were statistically analyzed (mean±SEM, n=8/group). (C) Kaplan–Meier analysis across the treatment groups in figure part B. (D, E) Flow cytometry analyses of CD8^+^ T cells (D) and IFN-γ^+^ CD8^+^ T cells (E) in subcutaneously transplanted tumors on day 19 of treatment. P<0.05 was considered statistically significant (n=8/group; two-tailed t-test).

The efficacy of ICBs is closely related to immune cell infiltration, especially CD8^+^ CTL infiltration.^3^ Several studies have shown that RT can remodel the tumor immune microenvironment and promote the infiltration and activation of CD8^+^ T cells.^11 31^ Therefore, the combination of RT and immunotherapy exhibits synergistic effects in many kinds of tumors. Our study found that inhibition of KDM4C could enhance the expression of the chemokine CXCL10 to promote the intratumoral infiltration and activation of CD8^+^ T cells. Accordingly, we surmised that SD70, an epigenetic inhibitor that explicitly targets KDM4C, could be combined with RT and immunotherapy to amplify these synergistic antitumor effects. To verify this hypothesis, we tested the impact of SD70, RT, and immunotherapy alone, in double combinations or in triple combination using C57BL/6 mice subcutaneously transplanted with Lewis cells (figure 5A). We found that SD70 alone could slow tumor growth compared with the vehicle, while combination with the anti-PD-L1 monoclonal antibody could further increase local tumor control and prolong survival in mice (figure 5B, C). Notably, although there was no significant difference between the SD70 plus RT group and the SD70 alone group on day 19, the tumor growth curves showed a significant synergistic effect thereafter (figure 5B). More importantly, triple therapy (SD70 plus RT and the anti-PD-L1 antibody) exhibited the highest tumor control rate and produced the longest survival time in mice (figure 5B, C).

By analyzing infiltrating CD8^+^ T cells in tumors by flow cytometry, we found that both monotherapies and dual combination therapies could increase the number of intratumoral CD8^+^ T cells and the proportion of cytotoxic IFN-γ^+^ CD8^+^ T cells, while the effect of the triple therapy was the most significant (figure 5D, E). Concurrently, we also conducted an in-depth evaluation of the safety of the combination therapy regimens. As shown in online supplemental figure S7A, combination therapy did not cause significant damage to the major organs of mice, such as the heart, liver, spleen, lungs, and kidneys. Moreover, analysis of the biochemical indexes of some major organs (such as those for liver function: TP, ALB, GLOB, ALT, ALP, Glu, Inorganic P, TBIL, CHOL, and ALB/GLOB; those for renal function: BUN, CRE, and BUN/CRE; those for cardiac function: CK and Ca^2+^; and that for pancreatic function: AMY) showed no significant abnormalities (online supplemental figure S7B). Our results indicated that the triple combination of SD70 and RT and the anti-PD-L1 monoclonal antibody achieved the strongest antitumor effect while remaining tolerable in lung cancer.

10.1136/jitc-2021-003716.supp8 Supplementary data

### CD8^+^ T cells and CXCL10 are required for antitumor immunity induced by the triple therapy in lung cancer

**Figure 6:**
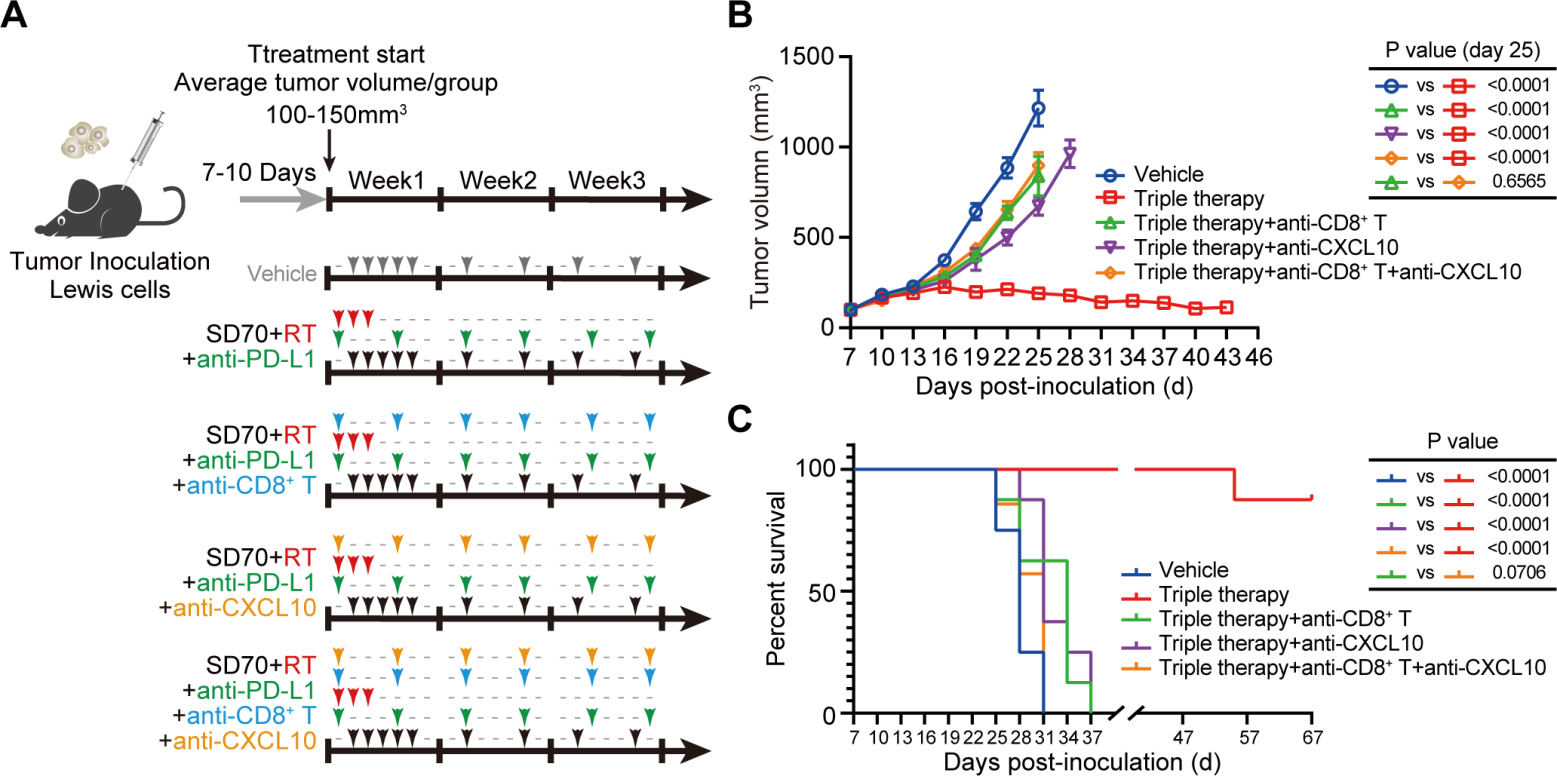
CD8^+^ T cells and CXCL10 are necessary for antitumor immunity induced by the triple therapy. (A) Schematic illustration of neutralization assays. Mice received one of five treatments on the indicated days: vehicle, triple therapy, triple therapy with an anti-CD8 neutralizing antibody, triple therapy with an anti-CXCL10 neutralizing antibody, and/or triple therapy with both the anti-CD8 and anti-CXCL10 neutralizing antibodies. (B) Mice (n=8/group) were inoculated with Lewis cells and given treatments as described in figure part A. The volumes of the subcutaneously transplanted tumors were recorded every 3 days, and the data on the 25th day were statistically analyzed (mean±SEM); p<0.05 was considered statistically significant). (C) Kaplan-Meier survival curves of mice bearing tumors in different treatment groups (n=8/group, Gehan-Breslow-Wilcoxon test).

To confirm the roles of CD8^+^ T cells and CXCL10 in the antitumor immune response following triple therapy administration, we depleted this population or cytokine in vivo and analyzed the effects on transplanted tumor growth and survival in the subcutaneous transplanted tumor model (figure 6A). Depletion of CD8^+^ T cells (p<0.001 vs triple therapy) or CXCL10 (p<0.001 vs triple therapy) greatly weakened the inhibitory effect of the triple therapy on the growth of transplanted tumors and shortened the survival time of mice (figure 6B, C). In addition, simultaneous depletion of CD8^+^ T cells and CXCL10 did not further reduce the antitumor effect of the triple therapy compared with the depletion of CD8^+^ T cells alone (figure 6B, C). These findings suggest that the antitumor immunity induced by the triple therapy is mainly achieved through the infiltration and activation of CD8^+^ T cells mediated by CXCL10.

## Discussion

In this study, we provided direct evidence that KDM4C is involved in antitumor immunity in lung cancer. Genetical or pharmacologic inhibition of KDM4C activated CXCL10 transcription by increasing the recruitment of the activated histone H3K36me3 at the CXCL10 promoter, which in turn increased the proliferation, migration, and activation of CD8^+^ T cells and delayed exhaustion in these cells in lung cancer. More importantly, we examined the synergistic effect of combining SD70, an epigenetic KDM4C-specific inhibitor, with RT and an anti-PD-L1 antibody and found that among the tested regimens, this triple therapy produced the best antitumor effects with tolerable toxicity in lung cancer.

The composition and functional status of immune cell subsets in the TME contribute to the ICB response. Epigenetic therapy has been shown to regulate various components of the TME and enhance antitumor immunity.^15 16^ In this study, we found that KDM4C, a key enzyme in epigenetic modification, was directly involved in regulating antitumor immunity in lung cancer. Analysis of the immune landscape following KDM4C inhibition revealed increased CD8^+^ T cell tumor infiltration and activation. These elevations in proliferation, migration, and direct killing coupled with delayed exhaustion in CD8^+^ T cells are in line with the strategy of making the tumor state ‘hotter’. Emerging evidence has shown that RT can kill tumor cells directly by inducing double-stranded DNA breaks in tumor cells and induce immunogenic cell death to recruit antigen-specific CD8^+^ T cells and enhance the antitumor immune response by releasing immunogenic tumor-associated antigens and chemokines.^32 33^ In our preclinical mouse model, we chose an 8 Gy ×3 regimen for RT because this regimen has been shown to have better immunogenicity than single 20 Gy and 6 Gy ×5 regimens.^31 34^ However, the best fractionation approach for RT is still an area of debate that warrants further investigation.

Previous studies have shown that the degrees of CD8^+^ T cell infiltration and activation are closely related to prognosis in many types of cancer.^6 35 36^ However, the underlying mechanisms are still elusive. CD8^+^ T cell infiltration requires chemokines to act as a ‘zip code’ to specify the trajectory of T cells. The chemokines CCL2, CCL3, CCl4, CCL5, CXCL9, CXCL10, and CXCL11 are known to be associated with T cell related tumor infiltration.^37^ CXCL9, CXCL10, and CXCL11 (ligands of CXCR3) are particularly critical because their expression is associated with prolonged disease-free survival in cancer patients.^6^ To explore the intricate mechanism of CD8^+^ T cell regulation by KDM4C inhibition, we performed RNA-seq and found that the cytokine–cytokine receptor signaling pathway was significantly enriched after SD70 treatment and that the expression of many chemokines was significantly upregulated. Further analysis found that the change in CXCL10 expression was the most significant. Western blotting and ELISA experiments also confirmed this result. More importantly, using ChIP-PCR, we revealed that H3K36me3 binding at the promoter region of CXCL10 was increased considerably after KDM4C inhibition, indicating that KDM4C directly regulates the transcriptional level of the CXCL10 gene in lung cancer. Furthermore, we uncovered that an anti-CXCL10 neutralizing antibody could partially reduce the increases in infiltration and activation and delay in exhaustion in CD8^+^ T cells following KDM4C inhibition. Furthermore, an anti-CXCL10 neutralizing antibody could partially eliminate the antitumor effect of the triple therapy. Therefore, we concluded that SD70-induced KDM4C inhibition increases the CXCL10 transcriptional level to mediate the increased intratumoral infiltration and activation of CD8^+^ T cells, which in turn enhances the efficacy of our triple therapy in lung cancer.

Antitumor immunity mediated by CD8^+^ CTLs is the cornerstone of immune-mediated tumor elimination and the determinant of ICB effectiveness.^3^ Our study provides direct evidence that KDM4C is involved in regulating CD8^+^ T cell infiltration and activity, which was the basis for combining SD70 with RT and immunotherapy. Importantly, we found that SD70 monotherapy or dual combination therapy, our triple therapy showed the most substantial tumor-inhibitory effect in a CD8^+^ T cell dependent and CXCL10-dependent manner. Furthermore, the triple therapy produced the most notable increases in the infiltration and functional activation of CD8^+^ T cells compared with the tested monotherapies and dual combination therapies. Additionally, to verify the safety and translational potential of this triple therapy, we comprehensively evaluated its safety in vivo. Our results clearly showed that the triple therapy did not cause significant damage to the major organs (heart, liver, pancreas, and kidneys) or abnormalities in peripheral blood biochemical tests, suggesting that the triple therapy is relatively safe and tolerable in vivo.

## Conclusions

**Figure 7:**
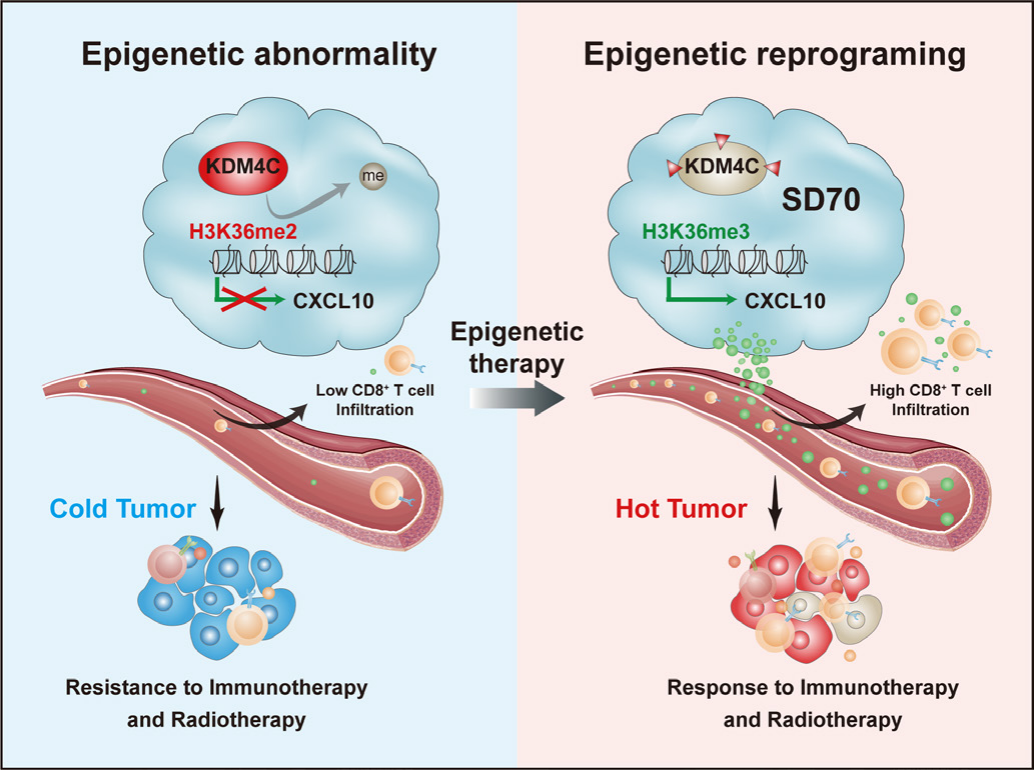
Schematic diagram showing the mechanism by which KDM4C is involved in antitumor immunity in lung cancer. Left panel: KDM4C is abnormally upregulated in lung cancer cells. By reducing the enrichment of the activated histone H3K36me3 at the promoter of CXCL10 and inhibiting CXCL10 expression, KDM4C reduces the infiltration and activation of CD8^+^ T cells, which converts the tumor to a ‘cold’ state that is insensitive to radiotherapy and immunotherapy. Right panel: targeted pharmacological inhibition of KDM4C using SD70 can reprogram the epigenetic state in a tumor, activate the expression of CXCL10, and enhance the recruitment and activation of CD8^+^ T cells, converting the tumor to a ‘hot’ state that is sensitive to radiotherapy and immunotherapy.

In summary, our results elucidate the role of KDM4C in regulating antitumor immunity. The aberrantly high expression of KDM4C in lung cancer inhibits CXCL10 transcription by reducing the enrichment of activated histone H3K36me3 at the CXCL10 promoter region, thus decreasing the infiltration and activation of CD8^+^ T cells. This converts the tumor into a ‘cold’ state that is resistant to RT and immunotherapy. The KDM4C-specific epigenetic inhibitor SD70 can reshape the tumor state, activate the expression of CXCL10, and enhance the recruitment and activation of CD8^+^ T cells, ultimately making tumors more responsive to RT and immunotherapy (figure 7).

## Data Availability

Data are available on reasonable request. Data used and analyzed in this study are available from the corresponding author on reasonable request.

## References

[1] Anandappa AJ, Wu CJ, Ott PA. Directing traffic: how to effectively drive T cells into tumors. Cancer Discov 2020;10:185–97. 10.1158/2159-8290.CD-19-0790 31974169 PMC7007384

[2] Topalian SL, Hodi FS, Brahmer JR, et al. Safety, activity, and immune correlates of anti-PD-1 antibody in cancer. N Engl J Med 2012;366:2443–54. 10.1056/NEJMoa1200690 22658127 PMC3544539

[3] Li J-Y, Chen Y-P, Li Y-Q, et al. Chemotherapeutic and targeted agents can modulate the tumor microenvironment and increase the efficacy of immune checkpoint blockades. Mol Cancer 2021;20:27. 10.1186/s12943-021-01317-7 33541368 PMC7863268

[4] Sharma P, Siddiqui BA, Anandhan S, et al. The next decade of immune checkpoint therapy. Cancer Discov 2021;11:838–57. 10.1158/2159-8290.CD-20-1680 33811120

[5] Bergamaschi C, Pandit H, Nagy BA, et al. Heterodimeric IL-15 delays tumor growth and promotes intratumoral CTL and dendritic cell accumulation by a cytokine network involving XCL1, IFN-γ, CXCL9 and CXCL10. J Immunother Cancer 2020;8:e000599. 10.1136/jitc-2020-000599 32461349 PMC7254133

[6] Wang D, Yu W, Lian J, et al. Th17 cells inhibit CD8^+^ T cell migration by systematically downregulating CXCR3 expression via IL-17A/STAT3 in advanced-stage colorectal cancer patients. J Hematol Oncol 2020;13:68. 10.1186/s13045-020-00897-z 32503584 PMC7275425

[7] van der Woude LL, Gorris MAJ, Halilovic A, et al. Migrating into the tumor: a roadmap for T cells. Trends Cancer 2017;3:797–808. 10.1016/j.trecan.2017.09.006 29120755

[8] Chen DS, Mellman I. Elements of cancer immunity and the cancer-immune set point. Nature 2017;541:321–30. 10.1038/nature21349 28102259

[9] van der Leun AM, Thommen DS, Schumacher TN. CD8^+^ T cell states in human cancer: insights from single-cell analysis. Nat Rev Cancer 2020;20:218–32. 10.1038/s41568-019-0235-4 32024970 PMC7115982

[10] Jhunjhunwala S, Hammer C, Delamarre L. Antigen presentation in cancer: insights into tumour immunogenicity and immune evasion. Nat Rev Cancer 2021;21:298–312. 10.1038/s41568-021-00339-z 33750922

[11] Meric-Bernstam F, Larkin J, Tabernero J, et al. Enhancing anti-tumour efficacy with immunotherapy combinations. Lancet 2021;397:1010–22. 10.1016/S0140-6736(20)32598-8 33285141

[12] Cabrera-Licona A, Pérez-Añorve IX, Flores-Fortis M, et al. Deciphering the epigenetic network in cancer radioresistance. Radiother Oncol 2021;159:48–59. 10.1016/j.radonc.2021.03.012 33741468

[13] Peng Q, Weng K, Li S, et al. A perspective of epigenetic regulation in radiotherapy. Front Cell Dev Biol 2021;9:624312. 10.3389/fcell.2021.624312 33681204 PMC7930394

[14] Lu Y, Chan Y-T, Tan H-Y, et al. Epigenetic regulation in human cancer: the potential role of epi-drug in cancer therapy. Mol Cancer 2020;19:79. 10.1186/s12943-020-01197-3 32340605 PMC7184703

[15] Zebley CC, Gottschalk S, Youngblood B. Rewriting history: epigenetic reprogramming of CD8^+^ T cell differentiation to enhance immunotherapy. Trends Immunol 2020;41:665–75. 10.1016/j.it.2020.06.008 32624330 PMC7395868

[16] Topper MJ, Vaz M, Marrone KA, et al. The emerging role of epigenetic therapeutics in immuno-oncology. Nat Rev Clin Oncol 2020;17:75–90. 10.1038/s41571-019-0266-5 31548600 PMC7254932

[17] Chen Y, Fang R, Yue C, et al. Wnt-induced stabilization of KDM4C is required for Wnt/β-catenin target gene expression and glioblastoma tumorigenesis. Cancer Res 2020;80:1049–63. 10.1158/0008-5472.CAN-19-1229 31888886 PMC7360480

[18] Cloos PAC, Christensen J, Agger K, et al. The putative oncogene GASC1 demethylates tri- and dimethylated lysine 9 on histone H3. Nature 2006;442:307–11. 10.1038/nature04837 16732293

[19] Berry WL, Janknecht R. KDM4/JMJD2 histone demethylases: epigenetic regulators in cancer cells. Cancer Res 2013;73:2936–42. 10.1158/0008-5472.CAN-12-4300 23644528 PMC3655154

[20] Jie X, Fong WP, Zhou R, et al. USP9X-mediated KDM4C deubiquitination promotes lung cancer radioresistance by epigenetically inducing TGF-β2 transcription. Cell Death Differ 2021;28:2095–111. 10.1038/s41418-021-00740-z 33558705 PMC8257660

[21] Lu Y, Li X, Liu H, et al. β-Trcp and CK1δ-mediated degradation of LZTS2 activates PI3K/AKT signaling to drive tumorigenesis and metastasis in hepatocellular carcinoma. Oncogene 2021;40:1269–83. 10.1038/s41388-020-01596-2 33420362 PMC7892348

[22] Huang Y, Yang X, Lu Y, et al. UBE2O targets Mxi1 for ubiquitination and degradation to promote lung cancer progression and radioresistance. Cell Death Differ 2021;28:671–84. 10.1038/s41418-020-00616-8 32901121 PMC7862231

[23] Jin C, Yang L, Xie M, et al. Chem-seq permits identification of genomic targets of drugs against androgen receptor regulation selected by functional phenotypic screens. Proc Natl Acad Sci U S A 2014;111:9235–40. 10.1073/pnas.1404303111 24928520 PMC4078819

[24] Takacs GP, Flores-Toro JA, Harrison JK. Modulation of the chemokine/chemokine receptor axis as a novel approach for glioma therapy. Pharmacol Ther 2021;222:107790. 10.1016/j.pharmthera.2020.107790 33316289 PMC8122077

[25] Canale FP, Ramello MC, Núñez N, et al. CD39 expression defines cell exhaustion in tumor-infiltrating CD8^+^ T cells. Cancer Res 2018;78:115–28. 10.1158/0008-5472.CAN-16-2684 29066514

[26] Kurachi M. Cd8+ T cell exhaustion. Semin Immunopathol 2019;41:327–37. 10.1007/s00281-019-00744-5 30989321

[27] Karin N. Chemokines and cancer: new immune checkpoints for cancer therapy. Curr Opin Immunol 2018;51:140–5. 10.1016/j.coi.2018.03.004 29579623

[28] Luo R, Firat E, Gaedicke S, et al. Cisplatin facilitates radiation-induced Abscopal effects in conjunction with PD-1 checkpoint blockade through CXCR3/CXCL10-mediated T-cell recruitment. Clin Cancer Res 2019;25:7243–55. 10.1158/1078-0432.CCR-19-1344 31506388

[29] Sen T, Rodriguez BL, Chen L, et al. Targeting DNA damage response promotes antitumor immunity through STING-mediated T-cell activation in small cell lung cancer. Cancer Discov 2019;9:646–61. 10.1158/2159-8290.CD-18-1020 30777870 PMC6563834

[30] Lercher A, Bhattacharya A, Popa AM, et al. Type I interferon signaling disrupts the hepatic urea cycle and alters systemic metabolism to suppress T cell function. Immunity 2019;51:1074–87. 10.1016/j.immuni.2019.10.014 31784108 PMC6926485

[31] Weichselbaum RR, Liang H, Deng L, et al. Radiotherapy and immunotherapy: a beneficial liaison? Nat Rev Clin Oncol 2017;14:365–79. 10.1038/nrclinonc.2016.211 28094262

[32] Marciscano AE, Ghasemzadeh A, Nirschl TR, et al. Elective nodal irradiation attenuates the combinatorial efficacy of stereotactic radiation therapy and immunotherapy. Clin Cancer Res 2018;24:5058–71. 10.1158/1078-0432.CCR-17-3427 29898992 PMC6532976

[33] Vanpouille-Box C, Alard A, Aryankalayil MJ, et al. DNA exonuclease TREX1 regulates radiotherapy-induced tumour immunogenicity. Nat Commun 2017;8:15618. 10.1038/ncomms15618 28598415 PMC5472757

[34] Dewan MZ, Galloway AE, Kawashima N, et al. Fractionated but not single-dose radiotherapy induces an immune-mediated abscopal effect when combined with anti-CTLA-4 antibody. Clin Cancer Res 2009;15:5379–88. 10.1158/1078-0432.CCR-09-0265 19706802 PMC2746048

[35] Byrne A, Savas P, Sant S, et al. Tissue-resident memory T cells in breast cancer control and immunotherapy responses. Nat Rev Clin Oncol 2020;17:341–8. 10.1038/s41571-020-0333-y 32112054

[36] Li X, Gruosso T, Zuo D, et al. Infiltration of CD8^+^ T cells into tumor cell clusters in triple-negative breast cancer. Proc Natl Acad Sci U S A 2019;116:3678–87. 10.1073/pnas.1817652116 30733298 PMC6397588

[37] Gordon-Alonso M, Hirsch T, Wildmann C, et al. Galectin-3 captures interferon-gamma in the tumor matrix reducing chemokine gradient production and T-cell tumor infiltration. Nat Commun 2017;8:793. 10.1038/s41467-017-00925-6 28986561 PMC5630615

