# Supplementary material for: Targeting KDM4C enhances CD8+ T cell mediated antitumor immunity by activating chemokine CXCL10 transcription in lung cancer
Source: J Immunother Cancer. 2022 Feb 4;10(2):e003716. doi: 10.1136/jitc-2021-003716 (PMC8819819; doi:10.1136/jitc-2021-003716)
Supplement: Supplementary data [file jitc-2021-003716supp001.pdf]

Figure legends

Supplementary Figure S1 KDM4C suppression inhibits the growth of transplanted tumors in C57BL/6 mice. (A) Lewis cells were transfected with a control or shKDM4C lentivirus and analyzed by Western blotting using the indicated antibodies (n=3). (B) Relative expression levels of KDM4C determined by RT-PCR (n=3). (C) Lewis cells stably expressing control or KDM4C shRNAs were seeded in 6-well plates. Cell numbers were counted every other day. $^{**}P < 0.01$ (n = 3). (D) Representative images of subcutaneous transplanted tumors in each group. (E) The IC50 values of SD70 in Lewis cells were obtained independently at least three times. (F) Western blotting was used to detect the effect of SD70 on KDM4C downstream gene expression (n=3). (G) Lewis cells treated with SD70 (1.5 μM) for 24 hours were seeded in 6-well plates. Cell numbers were counted every other day. $^{**}P < 0.01$ (n = 3). (H) Representative images of tumors in the indicated groups.

Supplementary Figure S2 Gating strategy for the analysis of tumor-infiltrating immune cells. Dead and live cells were distinguished by labeling with Zombie NIR, while the following antibody combinations were used to distinguish various immune cell populations in the live cell gate: $CD8^+$ T cells were characterized as $CD8^+$ $CD3^+$ $CD45^+$ cells. The different functional states of $CD8^+$ T cells were distinguished by the expression of surface molecules such as a proliferation marker (Ki67), cytotoxicity markers (IFN-γ, GZMB, Perforin and CD107a) and exhaustion markers (PD-1 and CD39). Treg cells were characterized as $FOXP3^+$ $CD45^+$ $CD4^+$ cells. Dendritic cells were characterized as $CD80^+$/$CD86^+$ $CD11c^+$ cells. Macrophages were characterized as $CD86^+$/$CD206^+$ $F4/80^+$ $CD11b^+$ cells. Gates were established with splenocytes from control group mice.

Supplementary Figure S3 Analysis of tumor-infiltrating immune cells by flow cytometry. The subcutaneously transplanted tumor experiment was carried out according to the method described in Figure 1A and 1C. A tumor was obtained and prepared into a single-cell suspension, and the composition of infiltrating immune cells in the tumor was analyzed by flow cytometry. (A) $CD3^+$ T cells, (B) $CD4^+$ T cells, (C) Treg cells, (D) B cells, (E) dendritic cells, and (F) macrophages. $P< 0.05$ was considered statistically significant.

Supplementary Figure S4 Construction of the *in vitro* conditioned culture model. (A) Schematic illustration of the construction process for the *in vitro* conditioned culture model. (B) Verification of the purity of $CD8^+$ T cells by flow cytometry. Data representative of three independently repeated experiments are shown.

Supplementary Figure S5 Targeting KDM4C enhances the expression and secretion of CXCL10. (A) The protein levels of CXCL10 were upregulated in KDM4C-depleted Lewis and human lung cancer cell lines (n=3). (B) The content of CXCL10 was measured by ELISA in multiple human lung cancer cell lines (A549, H1299 and H460). $^{**}P < 0.01$, $^{***}P < 0.001$ (n=3).

Supplementary Figure S6 The STING signal pathway and IFNα are not regulated by KDM4C. (A) A549 and Lewis cells were transfected with indicated siRNAs and analyzed by Western blotting using the indicated antibodies (n=3). (B) ELISA analysis of the exocrine level of IFN α (JL12191 & JL1203, Jianglai Biological) in A549 and Lewis cells. n. s. indicates no statistically significant difference ($P > 0.05$, $n = 3$).

Jie X, *et al. J Immunother Cancer* 2022; 10:e003716. doi: 10.1136/jitc-2021-003716

Supplementary Figure S7 Safety verification of various combined therapy modes. (A) Representative images of HE staining of the main organs (heart, liver, spleen, lungs and kidneys) of mice receiving the indicated treatment. Scale bar, 50μm; n=5/group. (B) Plasma was obtained from mice in each treatment group, and common peripheral blood biochemical indexes of mice were detected by an MNCHIP automatic biochemical analyzer (Pointcare M4). TP, ALB, GLOB, ALT, ALP, TBIL and CHOL were used to evaluate liver function; BUN, CRE and BUN/CRE were used to assess renal function; CK was used to evaluate cardiac function; AMY was used to evaluate pancreatic function; and no statistically significant changes were observed. The data were standardized by the Z-score, and a clustering heatmap was drawn. Abbreviations: ALB, albumin; TP, total protein; GLOB, globulin; $Ca2^{+}$, calcium cation; Glu, glucose; BUN, blood urea nitrogen; P, inorganic phosphorus; AMY, amylase; CHOL, cholesterol; ALT, alanine aminotransferase; TBIL, total bilirubin; ALP, alkaline phosphatase; CRE, creatinine; and CK, creatine kinase.

Jie X, *et al. J Immunother Cancer* 2022; 10:e003716. doi: 10.1136/jitc-2021-003716

Genes	Sequences (5'--3')
CXCL5	F: CTGCCCCTTCCTCAGTCATA<br>R: CAGACAGACCTCCTTCTGGTT
CXCL10	F: TCATCCTGCTGGGTCTGAGT<br>R: CCTATGGCCCTCATTCTCACT
CXCL1	F: CAATGAGCTGCGCTGTCAGT<br>R: CTATGACTTCGGTTTGGGTGC
CXCL2	F: CTGTCAATGCCTGAAGACCC<br>R: TGGCTATGACTTCTGTCTGGG
CCL20	F: GAAGCAGCAAGCAACTACGAC<br>R: GTTCACAGCCCTTTTCACCC
CCL2	F: GCTGACCCCAAGAAGGAATG<br>R: GTGCTTGAGGTGGTTGTGGA
CXCL3	F: AGTGCCTGAACACCCTACCA<br>R: GAGTGGCTATGACTTCTGTCTGG
KDM4C	F: TGAAGCAGCAGGTAGCGAGT<br>R: ATGAGCAGAGTGCAGATGGC
GAPDH	F: AGGTCGGTGTGAACGGATTTG<br>R: TGTAGACCATGTAGTTGAGGTCA

F, forward primer; R, reverse primer.

Supplementary Table S1 Sequences of primers used for qRT-PCR.

Genes	Primer	Sequences (5'--3')
CXCL10	Primer1	F: TCTGACTTCTGACTTCGTT<br>R: TCTCCAAAGTCAGCCAAT
	Primer2	F: TGTAAACCGAGGGCATTG<br>R: TAAAGCCATTTCCAGACT
	Primer3	F: TGCTTTGATTGTCTTCTT<br>R: ACCGGTACACTCCAGGCT
	Primer4	F: TCCCTTACTGAGGAGAAA<br>R: GAAACACCAAAGAAACAT
	Primer5	F: GAATTTCCTAAGAGTCCG<br>R: ACGTAACCCTAGCTATCC

F, forward primer; R, reverse primer.

Supplementary Table S2 Sequences of primers used for ChIP-PCR.
