# Supplementary figures and images for: Targeting KDM4C enhances CD8+ T cell mediated antitumor immunity by activating chemokine CXCL10 transcription in lung cancer
Source: J Immunother Cancer. 2022 Feb 4;10(2):e003716. doi: 10.1136/jitc-2021-003716 (PMC8819819; doi:10.1136/jitc-2021-003716)

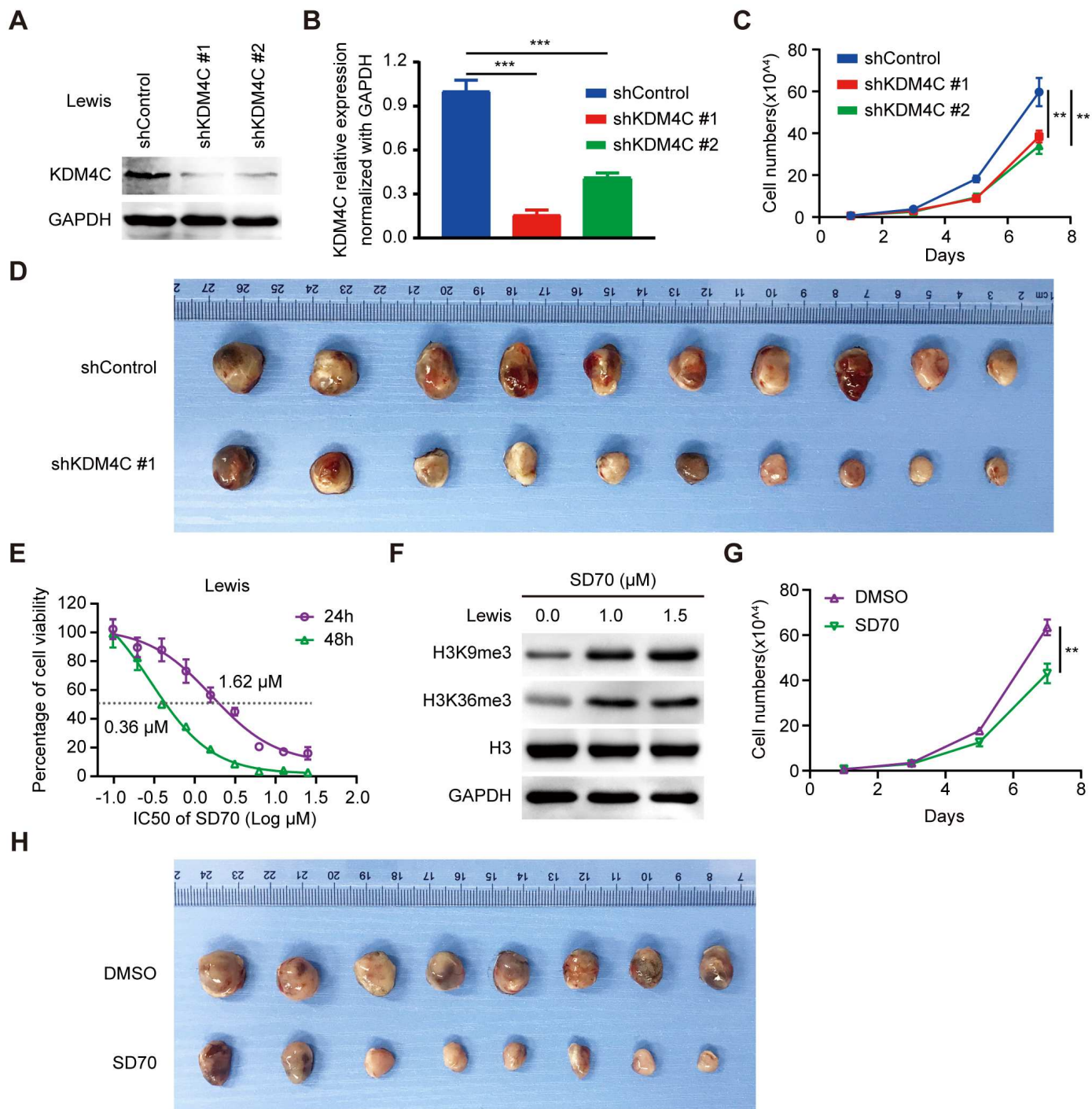

Supplement: Supplementary data [file jitc-2021-003716supp002.pdf]

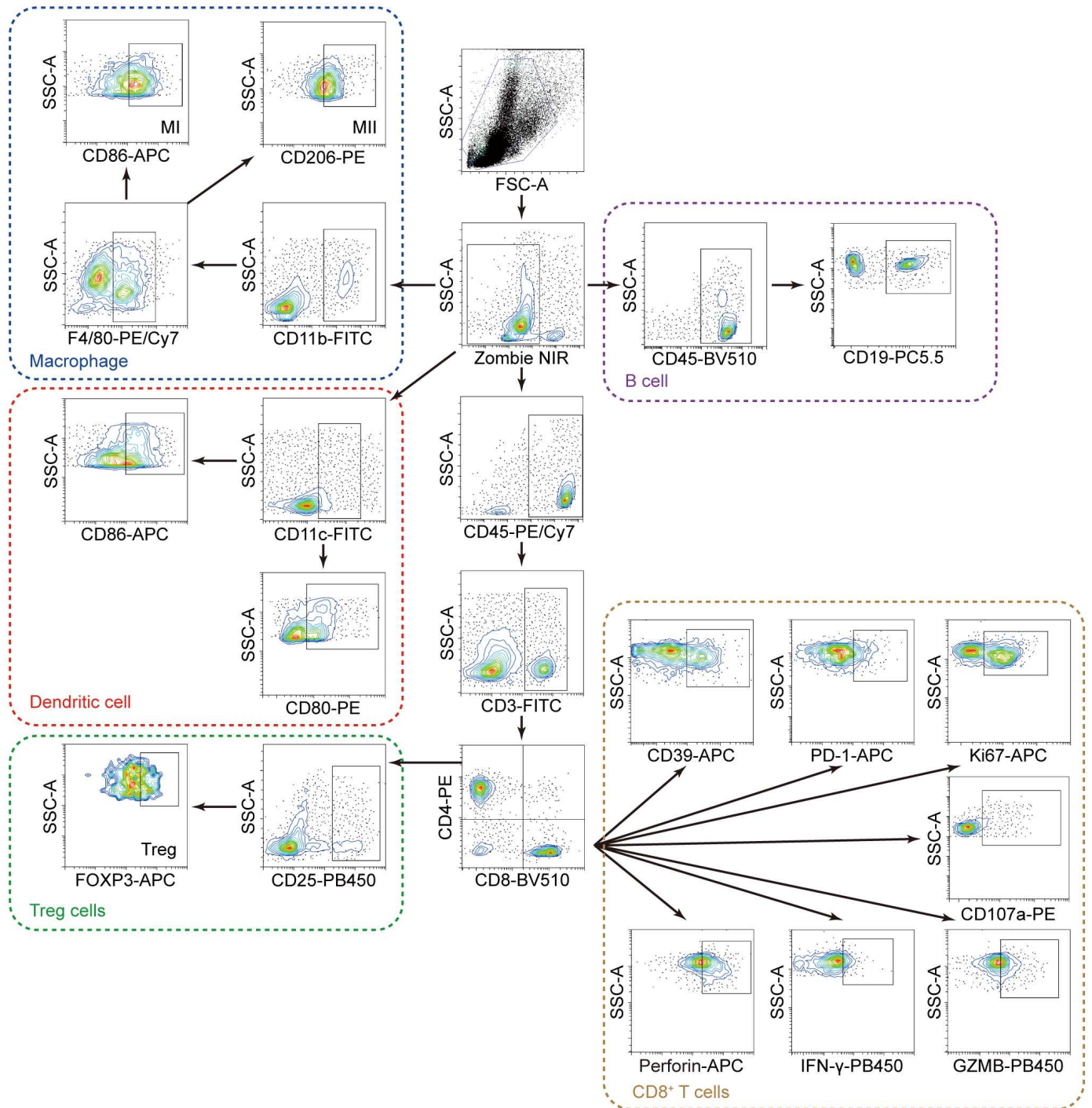

Supplement: Supplementary data [file jitc-2021-003716supp003.pdf]

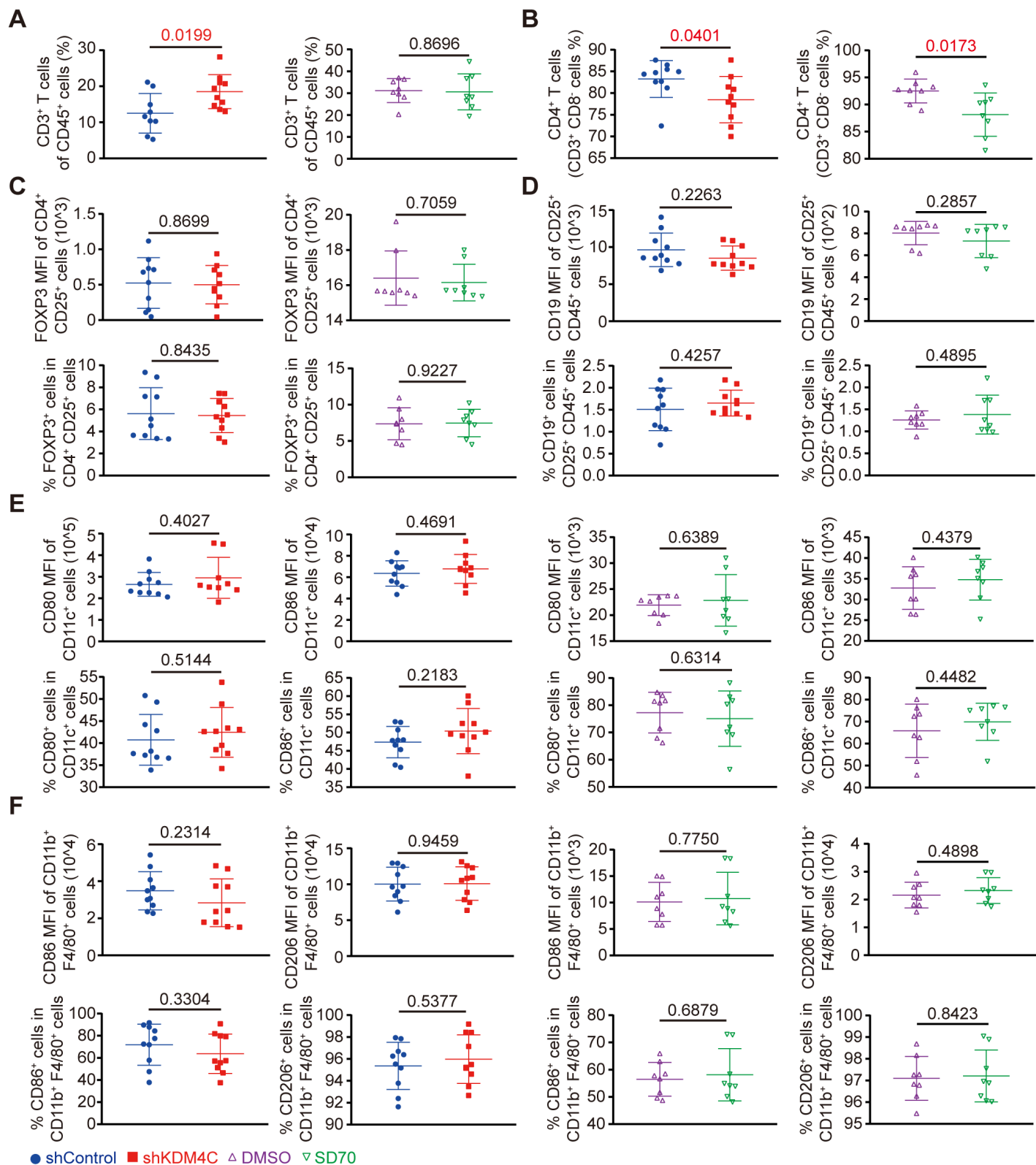

Supplement: Supplementary data [file jitc-2021-003716supp004.pdf]

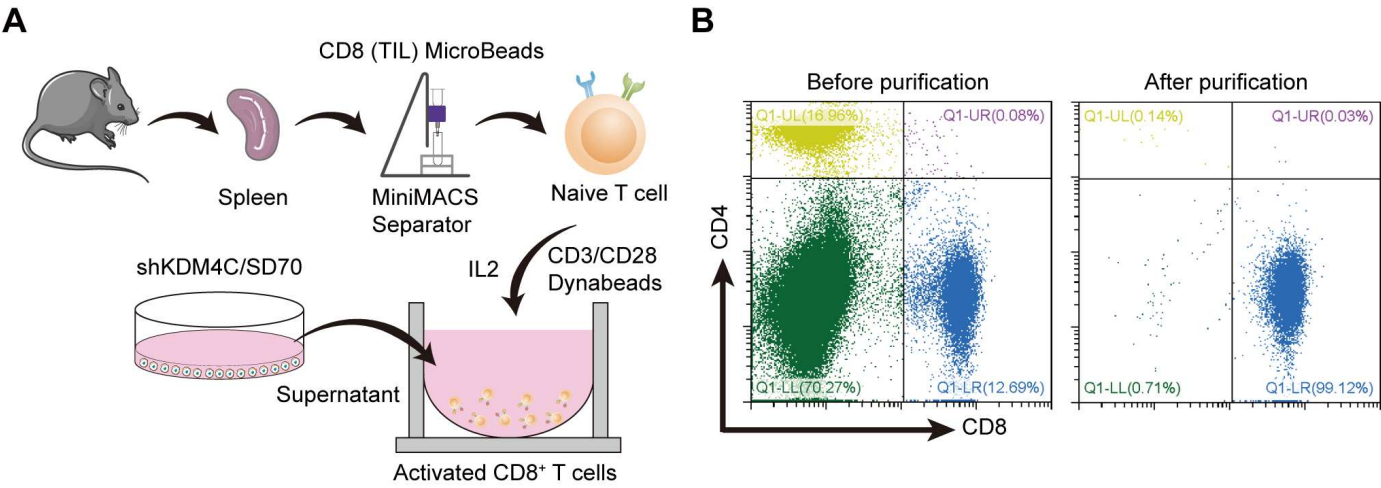

Supplement: Supplementary data [file jitc-2021-003716supp005.pdf]

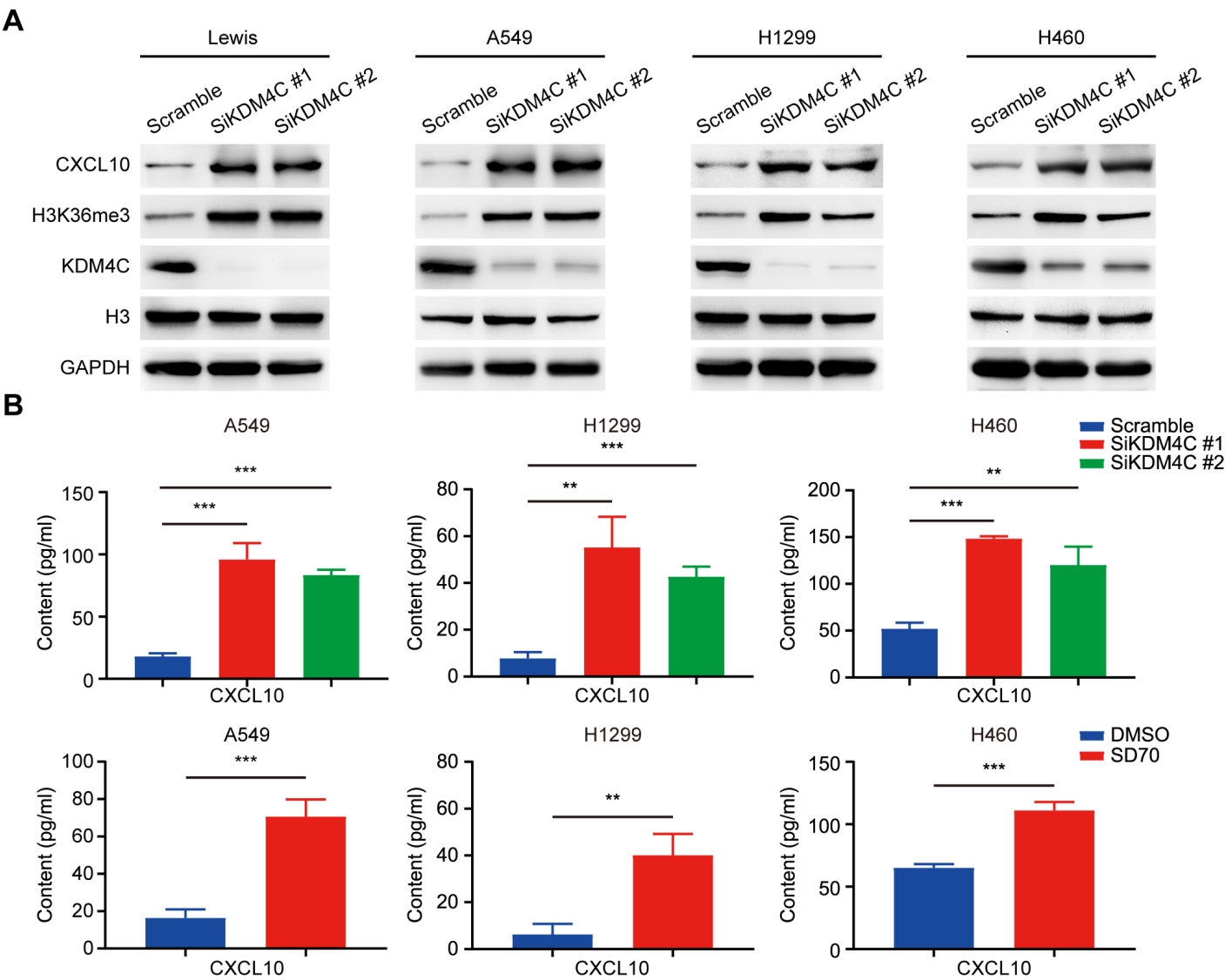

Supplement: Supplementary data [file jitc-2021-003716supp006.pdf]

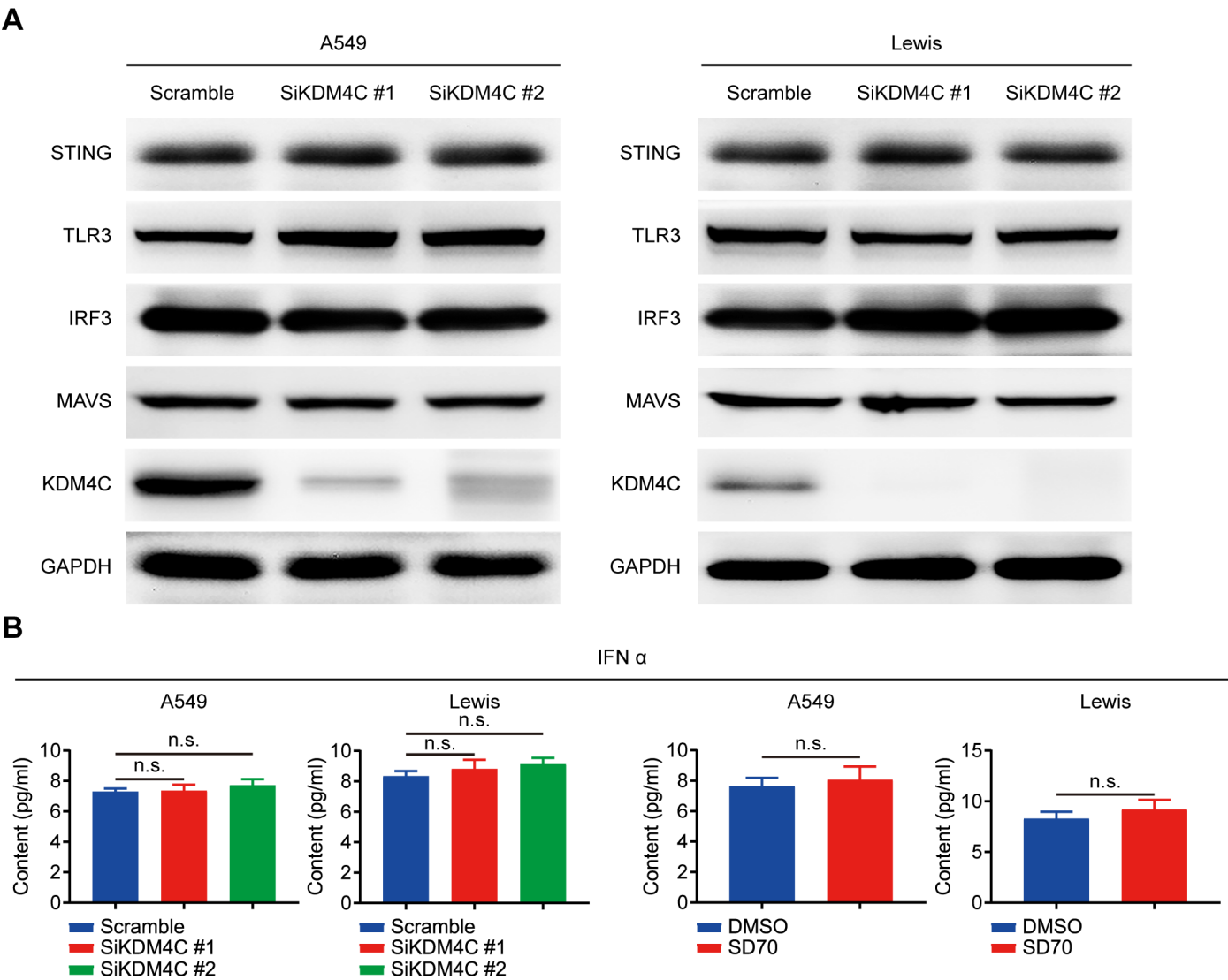

Supplement: Supplementary data [file jitc-2021-003716supp007.pdf]

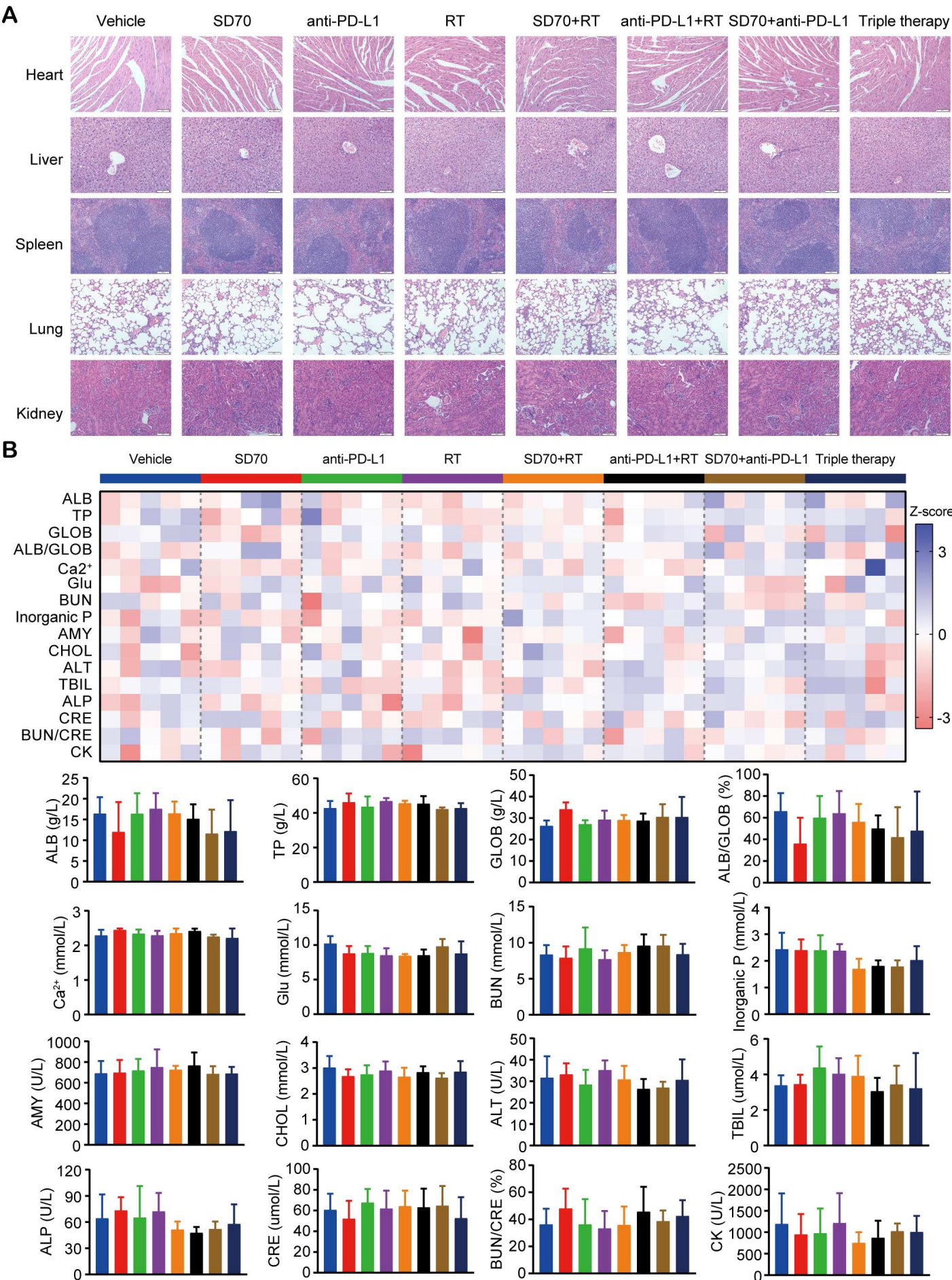

Supplement: Supplementary data [file jitc-2021-003716supp008.pdf]
